# Scanning the genomes of parents for imprinted loci acting in their un-genotyped progeny

**DOI:** 10.1038/s41598-018-36939-3

**Published:** 2019-01-24

**Authors:** Inga Blunk, Manfred Mayer, Henning Hamann, Norbert Reinsch

**Affiliations:** 1 Faculty of Agricultural and Environmental Sciences, University of Rostock, Justus-von-Liebig-Weg 6, 18059 Rostock, Germany; 2 Institute of Genetics and Biometry, Leibniz Institute for Farm Animal Biology (FBN), Wilhelm-Stahl-Allee 2, 18196 Dummerstorf, Germany; 3 State-Office for Geo-Information and Rural Development, Geodata-Center, Stuttgarter Straße 161, 70806 Kornwestheim, Germany

## Abstract

Depending on their parental origin, alleles at imprinted loci are fully or partially inactivated through epigenetic mechanisms. Their effects contribute to the broader class of parent-of-origin effects. Standard methodology for mapping imprinted quantitative trait loci in association studies requires phenotypes and parental origin of marker alleles (ordered genotypes) to be simultaneously known for each individual. As such, many phenotypes are known from un-genotyped offspring in ongoing breeding programmes (e.g. meat animals), while their parents have known genotypes but no phenotypes. By theoretical considerations and simulations, we showed that the limitations of standard methodology can be overcome in such situations. This is achieved by first estimating parent-of-origin effects, which then serve as dependent variables in association analyses, in which only imprinted loci give a signal. As a theoretical foundation, the regression of parent-of-origin effects on the number of *B*-alleles at a biallelic locus — representing the un-ordered genotype — equals the imprinting effect. The applicability to real data was demonstrated for about 1800 genotyped Brown Swiss bulls and their un-genotyped fattening progeny. Thus, this approach unlocks vast data resources in various species for imprinting analyses and offers valuable clues as to what extent imprinted loci contribute to genetic variability.

## Introduction

Genomic imprinting is an epigenetic mechanism in which the expression of genes is partially or entirely limited to one of the two inherited alleles. The effects of imprinted genes can be considered parent-of-origin effects (POEs) as they appear as phenotypic differences between heterozygotes depending on their parental allele origin^1^. The existence of imprinting is well established in plants, insects and mammals^2^. In avian species, imprinting is not confirmed as studies have resulted in contradictory results^3–6^. In plants, imprinting has been established to occur in maize^7–9^, *Arabidopsis thaliana*^10–14^, rice^15,16^, sorghum^17^ and castor beans^18^. The majority of imprinted genes in plants have been found in the endosperm and only a few are known to be expressed in the embryo^19^. In mammals, less than 1% of all genes are thought to be imprinted^1,20^. Imprinting has, however, crucial functions in stem cells, neuronal differentiation and growth^21^. It is thus responsible for a wide range of diseases in humans. Well known examples are Prader–Willi syndrome^22^ and Angelman syndrome^23^. In livestock, imprinted genes have considerable effects on the expression of agriculturally important traits. Variance-component analyses have revealed significant contributions of imprinted genes to the total genetic variance of traits in beef cattle and pigs^24–28^. With respect to pigs, a pioneering discovery was a purely paternally expressed polymorphism within the porcine IGF2, which was found to affect muscle mass and fat deposition traits^29,30^. The gene was detected by applying a line-cross design, where lines in a P_0_ generation (assumed to be fixed for the alleles *B* and *b* at the quantitative trait loci [QTLs], respectively) are crossed to create an F_1_ generation of individuals being heterozygous at these QTLs (summarised in Sandor and Georges^31^). Intercrossing these individuals generates an F_2_ population with equal frequencies of the four ordered genotypes *BB*, *Bb* and *bB*, *bb*. When the average phenotypes of *Bb* and *bB* can be statistically distinguished, i.e. a difference can be observed, the existence of an imprinted QTL (iQTL) is indicated. This, however, requires the knowledge of the parental allele origin (ordered genotype). Ordered genotypes are also needed in genome-wide association studies of POEs. For example, to account for POEs, Belonogova *et al*.^32^ added a vector *p* — containing the difference between the numbers of maternally and paternally derived *B*-alleles — as a second step in a model called GRAMMAR (rapid association using mixed model and regression^33^). Ordered genotypes also form the basis for genomic relationship matrices that account for imprinting^34^. To determine the ordered genotype of an offspring, conclusions on the parental allele origin can usually be drawn from the genotypes of its parents. However, if both parents are heterozygous, it is not possible to determine the offspring’s ordered genotype^32^. Then, if available, markers adjacent to the considered locus (haplotype information) may indicate the parental allele origin and thus the offspring’s ordered genotype. Furthermore, to detect iQTLs, phenotypic information is necessary in addition to ordered genotypes. This is a common issue in livestock, where the traits of interest are often measured in progeny which are often ungenotyped (e.g. milk production traits^35^) in contrast to genetically influential parents, such as bulls in dairy cattle. Therefore, there is a large amount of existing data that could be used for imprinting analyses.

To overcome the limitations of the currently applied methods of analysis for the detection of iQTLs, we propose a novel approach where estimated POEs (ePOEs) of parents are exploited to map imprinted genes expressed in their progeny. In the first step, phenotypic information can be summarised by applying a mixed *imprinting model* (first described in Blunk *et al*.^27^) that describes POEs as differences between two sex-specific transmitting abilities (TAs) for each parent. In the second step, the ePOEs are used as dependent variables in a model, where un-ordered genotypes of parents constitute the explanatory variable related to a particular fixed marker effect and a random polygenic effect accounts for the unconsidered genetic variability in the POEs. This novel approach does not require ordered genotypes or both genotypes and phenotypes from the same individuals. This principle is demonstrated in simulated data and is applied to empirical data consisting of genotyped Brown Swiss sires with ePOEs for slaughter traits. First, we provide theoretical justification by investigating the regression of POEs on the number of *B*-alleles at an imprinted locus in a random mating population.

## Theory

Here, we consider the regression of POEs on the number of *B*-alleles as a population parameter for a biallelic imprinted locus in a random mating (Fisher-Wright) population without selection and mutation. The alleles *B* and *b* have respective frequencies of *p* and *q* = 1 − *p*. Differences between the phenotypic means of the ordered genotypes ($\overline{BB}$, $\overline{Bb}$, $\overline{bB}$, $\overline{bb}$) are described by the additive effect *a* ($\overline{BB}$ − $\overline{bb}$ = 2*a*), the dominance effect *d* (0.5($\overline{Bb}$ + $\overline{bB}$) = *d*; assuming a centering of genotypes at the midpoint of the two homozygotes) and the imprinting effect *i *($\overline{Bb}$ − $\overline{bB}$ = 2*i)*, as described in Knott *et al*.^36^, Mantey *et al*.^37^ and the review by Lawson *et al*.^1^. Note that a somewhat different parameterization has been given by Spencer^38^. Parent-specific allele-substitution effects^39^ are given for male parents as ${\alpha }^{p}=a+(q\mbox{--}p)d+i=\alpha +i$ and for female parents as ${\alpha }^{m}=a+(q\mbox{--}p)d\mbox{--}i=\alpha \mbox{--}i$. The quantity $\alpha =a+(q\mbox{--}p)d$ is known as the allele-substitution effect in a standard Mendelian case^40^. In the presence of genomic imprinting, a locus exerts its effect on the offspring either under a male or a female expression pattern. In the former, the average phenotype of a parent’s offspring equals half of its breeding value ‘as father’ and in the latter, half of its breeding value ‘as mother’^24,25^. For the ordered genotypes *BB*, *Bb*, *bB* and *bb*, where the first allele is paternally derived, these breeding values can be expressed as functions of parent-specific substitution effects and allele-frequencies (Table 1). By following the definition in previous studies on variance components (e.g. Blunk *et al*.^27^), we define genotype-specific POEs as differences between the two sex-specific TAs for each genotype or, equivalently, as half the difference between both kinds of breeding values. For the four ordered genotypes, the POEs are 2*qi*, (*q* − *p*)*i*, (*q* − *p*)*i*, and −2*pi*, in the same order as above (Table 1).

**Table 1:** Ordered genotypes with their respective population genotype frequencies, number of *B*-alleles (gene count), breeding values (BVs) as father and as mother and parent-of-origin effects (POEs). The allele frequencies of *B* and *b* are denoted by *p* and *q* = 1 − *p*. The parameters *α* and *i* are the allele-substitution effect and the imprinting effect.

genotype	frequency	gene count	BV as father	BV as mother	POE
*BB*	*p* ^2^	2	2*q*(*α* + *i*)	2*q*(*α* − *i*)	2*qi*
*Bb*	*pq*	1	(*q* − *p*)(*α* + *i*)	(*q* − *p*)(*α* − *i*)	(*q* − *p*)*i*
*bB*	*pq*	1	(*q* − *p*)(*α* + *i*)	(*q* − *p*)(*α* − *i*)	(*q* − *p*)*i*
*bb*	*q* ^2^	0	− 2*p*(*α* + *i*)	− 2*p*(*α* − *i*)	− 2*pi*

For our purposes, we need to know the expectation and variance of the POEs in the population. The expectation is *E*(*POE*) = *p*^2^2*qi* + 2*pq*(*q* − *p*)*i* − *q*^2^2*pi* = 0, and the variance is given by *Var*(*POE*) = *p*^2^4*q*^2^*i*^2^ + 2*pq*(*q* − *p*)^2^*i*^2^ + *q*^2^4*p*^2^*i*^2^ = 2*pqi*^2^, which is equal to the imprinting variance at the locus, as derived by de Koning *et al*.^39^. Moreover, we are interested in the relationship between the number of *B*-alleles (gene count, *gc*), which has the expectation 2*p* and variance 2*pq*, and POEs. Their covariance can be derived as:

$$Cov(POE,gc)={p}^{2}(2\,-\,2p)2qi+2pq(1\,-\,2p)(q\,-\,p)i\,-\,{q}^{2}(0\,-\,2p)2pi=2pqi,$$

which translates into a correlation of *r* = 4*p*^2^*q*^2^*i*^2^. In summary, for a single imprinted locus, we have the covariance matrix:

$$Var[\begin{array}{c}POE\\ gc\end{array}]=2pq[\begin{array}{cc}{i}^{2} & i\\ i & 1\end{array}].$$

Finally, we arrive at the regression of the POEs on gene count, which is:

$$b=\frac{Cov(POE,\,gc)}{Var(gc)}=\frac{2pqi}{2pq}=i.$$

This final result shows that the regression of the POEs on the number of *B*-alleles of the same locus equals the imprinting effect. Next, we demonstrate how estimates of this population parameter can serve for marker-based imprinting analyses of simulated and real data.

## Data Background

### Simulated data

Importantly, POEs can be estimated for genotyped parents even when their phenotypes are not known, as it is often the case in livestock. In practice, ePOEs can first be obtained^24,25,27,28^ and, after a deregression step, be regressed on the number of *B*-alleles at a marker locus. In the light of the results for the single locus model, the resulting regression coefficient can then be interpreted as the imprinting effect of an iQTL in linkage and/or associated with the marker. Reliabilities of ePOEs are a prerequisite for deregression^41^. Only recently, their efficient calculation using mixed-model methodology has been described^27,28^.

We simulated two types of populations; the first comprised two generations, while the second contained three generations. Phenotypes were always available for the last generation only, while parents and grandparents remained without known phenotypes. The two-generation population was simulated to prove the applicability of the principle. One hundred unrelated parents were drawn from a base population. These parents were then inter-mated in a cross-classified manner, resulting in 100 × 100 full-sib families of size three. In this way, each parent was mated as father to all 100 parents (including itself), which acted as mothers. Vice versa, each parent was mated as mother to all 100 parents (including itself), which acted as father. This gave rise to 100 × 3 maternal half-sibs per parent. Thus, imprinted alleles were passed from each parent to progeny with both of the two possible parental expression patterns.

In the three-generation pedigree, sources of family information contributed to the ePOEs of the individuals in generations one and two. These sources were the parent-average (PA), records of the final progeny and records of the progeny’s final progeny. The naïve utilization of ePOEs as dependent variables would negatively influence the outcome of association studies as shown for breeding values in Ekine *et al*.^42^. Thus, deregressed and weighted ePOEs, free from the influence of the PA, were needed. The three-generation population was simulated to illustrate the effects of the PA-correction, deregression and weighting. Ten grandparents were randomly chosen from the base population and produced 100 full-sib families, whose variable size was chosen from a Poisson distribution with a mean of 5.0. This led to 514 parents in the second generation, which were again inter-mated in a cross-classified manner. The number of full-sibs per family in the third generation was also Poisson distributed with a mean of 5.0. Only a fraction of all possible progeny (3%) with phenotypes was retained so that 41,273 records remained for the analysis. The full-sib families contained one to four phenotyped offspring each.

In both population types, 15 mutually unlinked marker genotypes were simulated, where five were in linkage disequilibrium (LD) with QTLs. The LD was created by first drawing the QTL alleles from a binomial distribution depending on their allele frequencies. The alleles of the adjacent markers were then drawn from a conditional distribution, where an initial LD of 0.1 among founders was assumed. The first QTL at marker locus two (minor allele frequency [MAF] = 0.4) was chosen to be biparentally expressed (Mendelian QTL) contributing 30% to a total additive genetic variance of 1.35. A purely paternally and a purely maternally expressed QTL each contributed 5% to the total additive genetic variance and 25% to the total imprinting variance of 0.27. These were linked to the marker loci five (MAF = 0.5) and eight (MAF = 0.5). The marker loci 11 (MAF = 0.5) and 14 (MAF = 0.5) were both linked to partially imprinted QTLs with opposite POEs, which contributed 30% to the total additive genetic variance and 25% to the total imprinting variance. A residual variance of 2 generated a heritability of 0.40 for the simulated trait, and the imprinting variance overall accounted for 20% of the total additive genetic variance. The trait was created by adding additive and imprinting effects according to the simulated QTL genotypes. All genetic distances between associated markers and QTLs were set to 10 centiMorgans in the base population. For each of the two populations, 100 repetitions were simulated by drawing new genotypes and residual effects without altering family sizes or pedigree structures.

Next, we compared the results for imprinted and un-imprinted (Mendelian) QTLs under both our new and the traditional approach. Therefore, four different kinds of analyses, labelled 1A, 1B, 2A and 2B, were applied to the two-generation data. First, in analyses 1A and 1B, we made use only of the un-ordered genotypes and ePOEs and TAs of the 100 parents. Second, in two consecutive association analyses (2A, 2B), we assumed that the phenotypes and ordered genotypes of the 30,000 offspring were available. To find associations with imprinted loci, in analysis 2A an adapted version of a measured genotype model^43^ was applied. Analysis 2B considered additive effects only by neglecting the imprinting effect.

Finally, to illustrate the effects of the PA-correction, deregression and weighting, the ePOEs from the three-generation data were used for association analyses. In analysis 3A, ePOEs were left completely untreated, while in 3B they were deregressed and PA-corrected, and in 3C, the ePOEs were additionally weighted. A summary of the analyses is provided in Table 2.

**Table 2:** Overview of analyses using different models and genotypic information. The dependent variable *y* is either the parent-of-origin effect of a parent (ePOE), the transmitting ability of a parent (TA) or the observed phenotype of an offspring. In analysis 3, the ePOEs were deregressed, parent-average (PA) corrected (3B), and weighted (3C); *x*_*a*_ is the gene content of an un-ordered genotype (parental allele origin unknown) at a marker locus; *x*_*p*_ has the values of 0, 1, −1, and 0 for the ordered genotypes *BB*, *Bb*, *bB* and *bb* (parental allele origin known); *b*, *b*_*a*_, and *b*_*p*_ are the regression coefficients; the random variables *u*, *g*_*s*_ (effect of the father’s gamete) and *g*_*d*_ (effect of the mother’s gamete) account for the stratification of the population into families; *μ* is the general mean and *e* is the residual.

analysis	model	dependent variable (*y*)	genotypic information
**1A**	*y* = *μ* + *bx*_*a*_ + *e*	ePOEs	un-ordered
**1B**	*y* = *μ* + *bx*_*a*_ + *e*	TAs	un-ordered
**2A**	*y* = *μ* + *b*_*a*_x_*a*_ + *b*_*p*_*x*_*p*_ + *g*_*s*_ + *g*_*d*_ + *e*	observed phenotype	un-ordered and ordered
**2B**	*y* = *μ* + *b*_*a*_*x*_*a*_ + *g*_*s*_ + *g*_*d*_ + *e*	observed phenotype	un-ordered
**3A**	*y* = *μ* + *bx*_*a*_ + *u* + *e*	ePOEs	un-ordered
**3B**	*y* = *μ* + *bx*_*a*_ + *u* + *e*	ePOEs (deregressed, PA-corrected)	un-ordered
**3C**	*y* = *μ* + *bx*_*a*_ + *u* + *e*	ePOEs (deregressed, PA-corrected, weighted)	un-ordered

### Brown Swiss data

The ePOEs and parental TAs as well as their reliabilities were derived from an imprinting analysis of Brown Swiss cattle slaughterhouse data published in detail in Blunk *et al*.^27^. They were derived for up to 428,710 sires and dams based on the routinely recorded performance of their progeny, which were up to 173,051 fattening bulls (exact numbers vary from trait to trait). The imprinting variance contributed a significant proportion to the total genetic variance in the net body weight (BW) gain (carcass weight divided by age [g/d]), carcass muscularity and carcass fatness. While muscularity was described using five monetary grades reflecting price differences, fatness was categorised by scores ranging from 1 (lean) to 5 (very fat). Assuming these traits to be normally distributed, ePOEs were first generated applying a linear *imprinting model* (these traits are subsequently indicated with the subscript *L*). Then, ePOEs were generated applying a threshold *imprinting model* assuming the traits to be binomially distributed (subscript *T*). As data were routinely obtained, animal care guidelines were not obtained.

Upon agreement of all involved organisations, genotypes were made available from the central genome database, which is maintained for breeding purposes at LKV Bayern in Munich. Data retrieval was assisted by the Institute for Animal Breeding at the Bavarian State Research Centre for Agriculture. After quality control, 37,443 genotypes remained for 1,857 sires for the net BW gain and 37,433 genotypes remained for 1,831 sires for the muscularity and fatness traits.

The genome was scanned via single marker regression, where ePOEs, defined as deviations of the TAs as dam from the TAs as sire, were regressed on their marker gene counts individually. With respect to the TAs (derived from the reduced *imprinting model*), both kinds (TAs as sire and TAs as dam) were used as dependent variables. Both ePOEs and TAs were PA-corrected, deregressed and weighted adapting the approximate method published in Garrick *et al*.^41^ for breeding values. When their deregressed reliabilities were <3%, they were discarded. With regard to ePOEs, 1,793 remained for the net BW gain with an average reliability of 25.86%; 1,033 remained for muscularity_**(L)**_ with an average reliability of 9.73%; 1,720 remained for muscularity_**(T)**_ with an average reliability of 14.01%; 1,277 remained for fatness_**(L)**_ with an average reliability of 10.63%; and 1,649 remained for fatness_**(T)**_ with an average reliability of 12.51%. As discussed in detail later, the sensitivity to *c*, which was chosen to calculate the weighting of ePOEs and which defines the proportion of the imprinting variance not captured by markers^41^, was not particularly high in the simulation study. Therefore, *c* was limited to 0.1, 0.5 and 0.8. These values were only applied during the genome scan for ePOEs on the net BW gain to investigate the effect of changing *c* within a real data framework. A *c* of 0.1 was chosen to analyse the ePOEs on all other traits.

## Results and Discussion

### **Simulated data**

**Figure 1:**
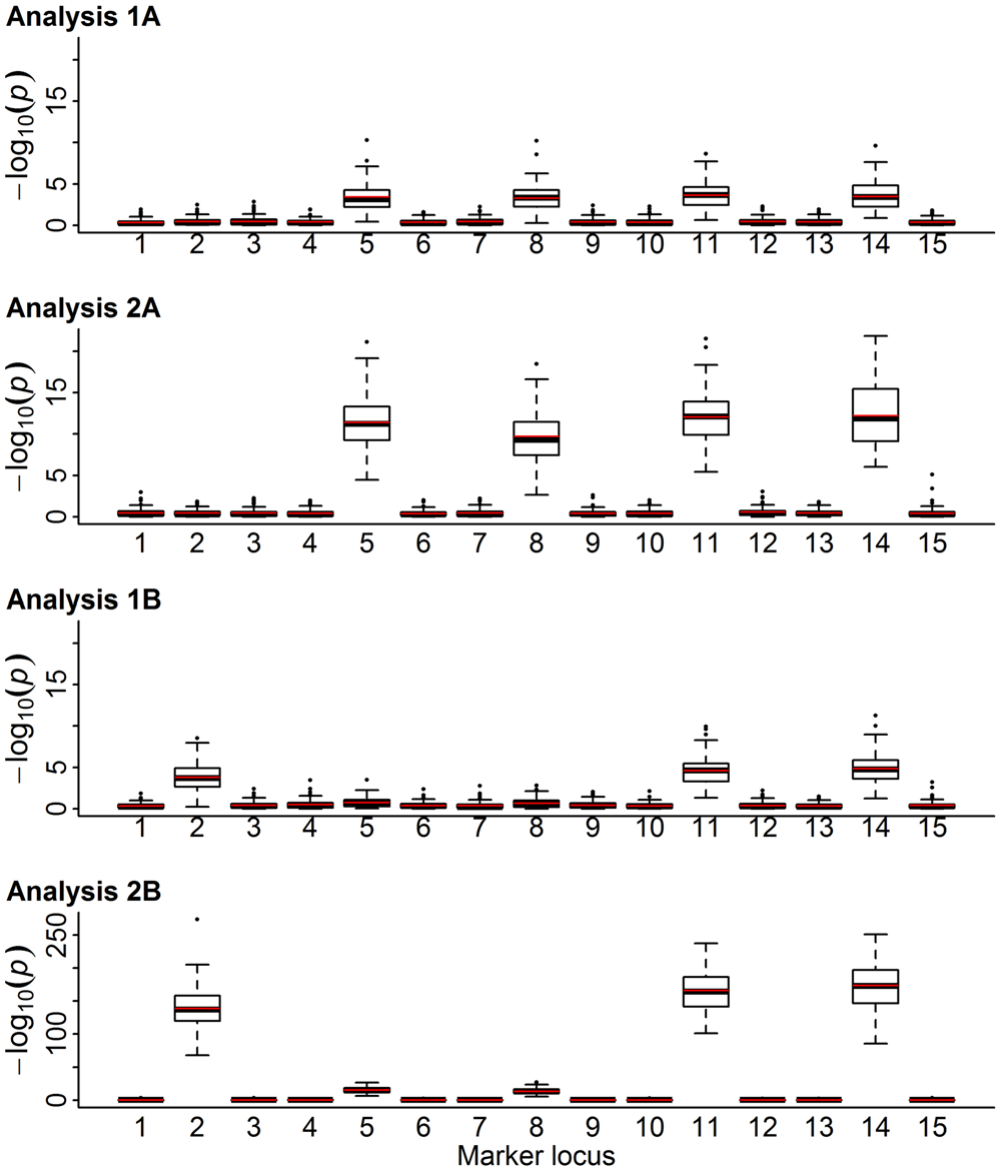
Boxplots of the −log_10_ *p*-values and their means (red line) for 15 markers in simulated two-generation population (with 100x replications) from different kinds of analyses: Estimates of the parent’s parent-of-origin effects (analysis 1A) and transmitting abilities (analysis 1B) were regressed on their un-ordered genotype; phenotypes of offspring were regressed on their own ordered genotype (analysis 2A) and un-ordered genotype (analysis 2B). Quantitative trait loci (QTLs) showed biparental, paternal, maternal, partial paternal and partial maternal expression patterns at markers 2, 5, 8, 11 and 14, respectively; no other markers were associated with any QTL.

To investigate whether ePOEs are suitable to detect iQTLs, they were used as dependent variables regressed on the gene counts of 100 unrelated parents in the simulated two-generation data (analysis 1A). At the fully imprinted marker loci (five and eight), the average *p*-values ranged from *p* = 0.012 to *p* = 0.025, indicating the presence of imprinted loci (1A in Fig. 1; Table 3). The signals at the marker positions 11 and 14 (linked to partially expressed iQTLs) were visible with average *p*-values of 0.010, respectively. In contrast, the mean estimated effect at marker locus two (linked to a purely Mendelian QTL) did not significantly deviate from zero. As expected, the same pattern of signals was observed when phenotypes and ordered marker genotypes of offspring were used in a measured genotype model to detect iQTLs in analysis 2A (2A in Fig. 1; Table 3). Together, these results provide proof of principle that associations with iQTLs can be detected by regressing ePOEs on un-ordered genotype information. In comparison to ePOE-analysis 1A, higher average signals (in terms of −log_10_ *p*-values) indicated the positions of iQTLs (2A in Fig. 1) when phenotypes of offspring served as the dependent variable (analysis 2A). However, ordered marker genotypes of 30,000 individuals with records were necessary to observe this result, whereas un-ordered genotypes of only 100 parents without records were used in analysis 1A. Therefore, using ePOEs as dependent variables constitutes a suitable alternative to detect iQTLs especially when ordered marker genotypes are not available for individuals with phenotypes or phenotypes are not observed for the individuals with marker genotypes. The ePOEs and reliabilities can be directly estimated for parents by applying the reduced *imprinting model*^27^. This model is also available in a sire-maternal grandsire version, which is particularly helpful when there are large data sets. This was demonstrated in Blunk *et al*.^28^, where more than 1.3 Mio records of Simmental fattening bulls were analysed.

**Table 3:** Average *F*-statistics (*F*) and *p*-values (*p*) for all analyses and markers linked to quantitative trait loci (QTLs) with biparental (Men), paternal (Pat), maternal (Mat), partial paternal (Pat/Men) and partial maternal (Mat/Men) expression patterns. For markers not linked to QTLs (others), ranges are given. Subscripts *imp* and *add* indicate separate tests related to imprinting, or Mendelian effects, while *mkr* indicates an unspecified single effect marker test from model 2B. Estimates of the parent’s parent-of-origin effects (1A) and transmitting abilities (1B) were regressed on their un-ordered genotype; phenotypes of offspring were regressed on their own ordered (2A) and un-ordered (2B) genotype. Estimated parent-of-origin effects were analysed untreated (3A), deregressed and corrected for parent average (3B), and deregressed, corrected for parent-average and weighted (3C). For 3C, the average weighting parameter *c*, its standard deviation (*sd*) between repeated simulations and the average log-likelihood is also given.

analysis		2	5	8	11	14	others
		Men	Pat	Mat	Pat/Men	Mat/Men	no
1A	*F*_*imp*_	1.137	14.21	13.48	15.17	14.41	0.798–1.224
	*p*_*imp*_	4.5e-01	1.2e-02	2.5e-02	1.0e-02	1.0e-02	4.6e-01–5.4e-01
1B	*F*_*add*_	15.93	2.153	1.913	20.24	21.47	0.743–1.358
	*p*_*add*_	1.1e-02	3.3e-01	3.7e-01	2.0e-03	2.0e-03	4.7e-01–5.5e-01
2A	*F*_*add*_	634.3	66.22	58.98	755.6	792.8	0.886–1.247
	*p*_*add*_	7.9e-7	3.3e-09	5.0e-08	9.6e-104	2.8e-88	4.4e-01–5.4e-01
2A	*F*_*imp*_	1.068	48.28	40.69	51.29	51.97	0.930–1.481
	*p*_*imp*_	4.7e-01	3.8e-07	2.6e-05	5.8e-08	4.4e-08	4.1e-01–5.1e-01
2B	*F*_*mkr*_	634.3	66.35	59.00	757.3	794.0	0.888–1.249
	*p*_*mkr*_	7.9e-71	3.4e-09	4.4e-08	7.9e-104	4.1e-88	4.3e-01–5.4e-01
3A	*F*_*imp*_	1.647	25.84	22.91	27.27	33.39	0.806–1.353
	*p*_*imp*_	4.2e-01	3.0e-02	4.9e-02	1.8e-02	1.7e-02	4.5e-01–5.6e-01
3B	*F*_*imp*_	1.664	26.10	23.19	27.43	33.68	0.813–1.341
	*p*_*imp*_	4.0e-01	2.6e-02	4.6e-02	1.7e-02	1.8e-02	4.5e-01–5.6e-01
3C	*F*_*imp*_	1.632	26.08	23.29	27.48	33.67	0.803–1.379
	*p*_*imp*_	4.1e-01	2.7e-02	4.6-e02	1.6e-02	1.7e-02	4.5e-01–5.6e-01
	*c*	0.328	0.340	0.311	0.328	0.324	0.316–0.327
	*c*_*sd*_	0.332	0.348	0.332	0.343	0.333	0.327–0.334
	*logL*	341.340	353.061	351.759	353.671	356.445	340.967–341.339

When, in contrast to ePOEs, estimated TAs were used as dependent variables (analysis 1B), mean *p*-values ranging from 0.002 to 0.011 indicated QTLs at all loci with a Mendelian component, i.e. purely Mendelian and partially imprinted loci (1B in Fig. 1; Table 3). The mean effects estimated for the two fully imprinted markers did not significantly deviate from zero, with mean *p*-values of 0.328 and 0.369. This demonstrates that TAs, or, equivalently, breeding values, are only suitable to detect QTLs contributing to Mendelian genetic variation but not to detect fully imprinted loci, nor do such analyses offer any clue as to the imprinted nature of partially imprinted QTLs. The same is true for the model for observed phenotypes in analysis 2B, that did not consider imprinting, since only an additive effect was included. At all marker loci, the mean test statistics indicated the existence of QTLs without the possibility to distinguish Mendelian and imprinted loci, exactly as was the case with TAs (Table 3).

**Figure 2:**
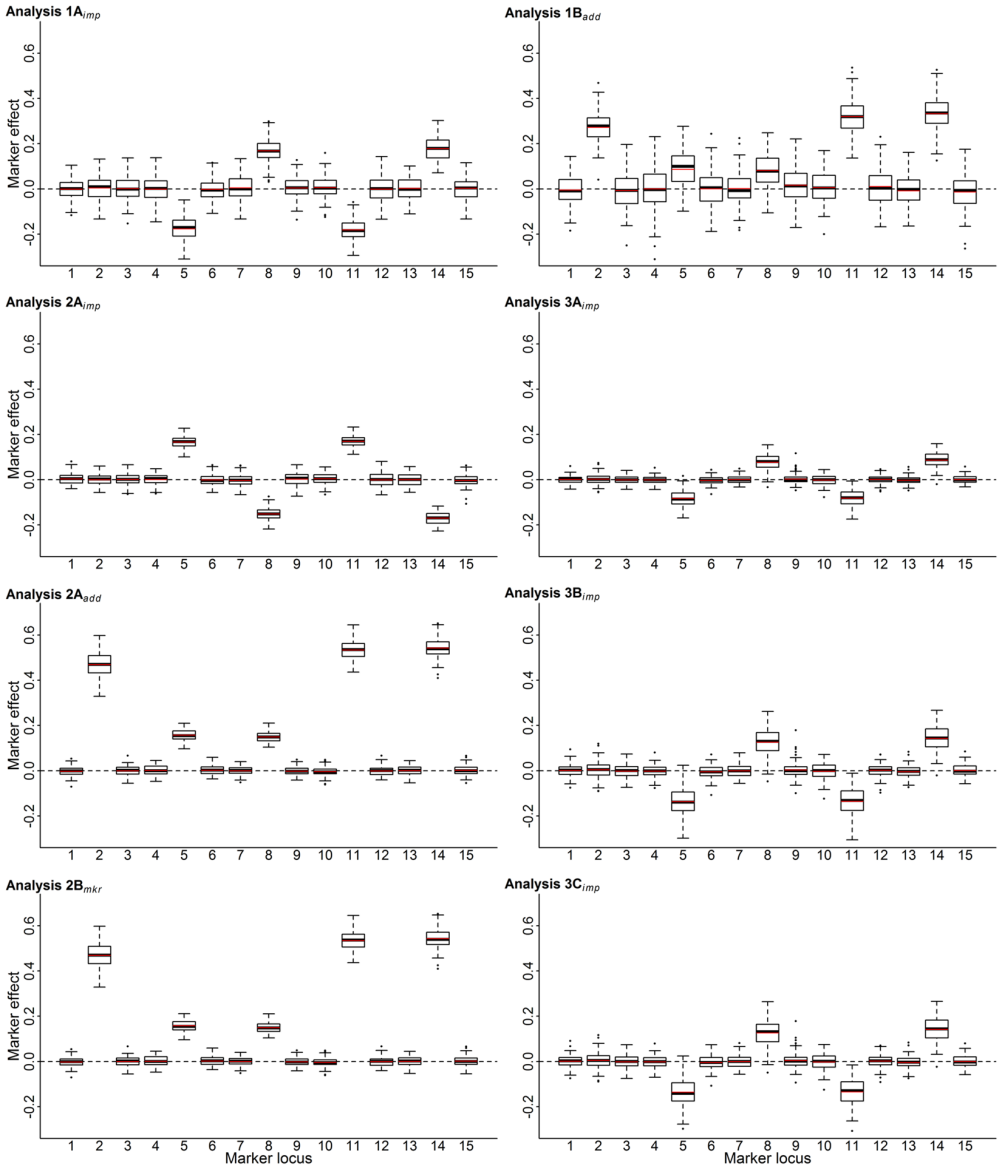
Boxplots of the marker effects and their means (red line) for all analyses and markers linked to quantitative trait loci (QTLs) with biparental (marker 2), paternal (marker 5), maternal (marker 8), partial paternal (marker 11) and partial maternal (marker 14) expression patterns. Subscripts *imp* and *add* indicate separate tests related to imprinting, or Mendelian effects, while *mkr* indicates an unspecified single effect marker test from model 2B. Estimates of the parent’s parent-of-origin effects (1A) and transmitting abilities (1B) were regressed on their un-ordered genotype; phenotypes of offspring were regressed on their own ordered (2A) and un-ordered genotype (2B). Estimated parent-of-origin effects were analysed untreated (3A), deregressed and corrected for parent average (3B), and deregressed, corrected for parent-average and weighted (3C).

With regard to the estimation of marker effects, estimates were of similar magnitude at all marker loci when ePOEs were used as dependent variables in analysis 1A and when the model for phenotypes included an imprinting effect in analysis 2A (Fig. 2; Supplementary Table S1). Note, however, that the sign was reversed. The sign of ePOEs depends on their definition in the *imprinting model*^27^, which can be either the genetic effect as father minus the genetic effect as mother or the reverse. Neither the estimation of the imprinting variance nor the detection of iQTLs depends on the sign of that difference as it has no effect on the absolute value of the marker effect and its estimate. When, in contrast to ePOEs, estimated TAs were used as dependent variables (analysis 1B), the estimates were about half the magnitude of those observed when the model for phenotypes included an additive effect in analyses 2A and 2B (Fig. 2; Supplementary Table S1). Since a TA equals half the breeding values, this was expected.

To illustrate the effects of the PA-correction, deregression and weighting, the ePOEs from the three-generation data were used in analyses 3A, 3B and 3C. In analysis 3A, ‘untreated’ ePOEs of parents were regressed on their own un-ordered marker genotypes. Again, the mean *p*-values indicated that ePOEs can be used to detect imprinted loci, although a further loss of power must be noted in comparison to analysis 1A (Table 3). The loss of power may be explained by a loss of LD between the QTLs and their markers due to more recombination events since the base generation until alleles are transmitted to the last (third) generation. With an initial LD of 0.1 among founders, their distance was large (10 centiMorgans), so the LD decreased rapidly from one generation to the next. As the ePOEs of individuals in generation two showed an undesired regression to their PA with an average reliability of 0.80, the ePOEs were deregressed and PA-corrected in analysis 3B. In comparison to the results in analysis 3A, only minor changes in the mean *F-*values and *p*-values were observed (Table 3). In contrast, the deregression and PA-correction increased the mean estimates of marker effects by almost one-third so that they approximated the estimates observed when ePOEs from the two-generation data were used as dependent variables in analysis 1A (Fig. 2; Supplementary Table S1). Due to their heterogeneous variance, ePOEs were additionally weighted in analysis 3C. With regard to the test statistics, no particular differences could be observed in comparison to analysis 3B (Table 3). Moreover, neither the mean marker effect estimates nor their variation and standard errors differed (Fig. 2; Supplementary Table S1). To calculate the weighting of ePOEs, a *c*-parameter was needed. An examination of the mean *c*-parameters across all replications demonstrated that a grid-search, which was performed to choose a suitable *c*-parameter, did not favour *c*-values in a certain range because no particular differences could be observed between markers. However, the standard deviations reflected strong fluctuations among replications. The log-likelihoods changed little in relation to the development of *c* (Table 3). This indicates a flat log-likelihood function and thus a low sensitivity to *c*. According to Garrick *et al*.^41^, this sensitivity depends on the heterogeneity of the information content in the data. Thus, due to the straightforward simulation design, a low sensitivity could have been expected from the outset.

To summarise, no particular changes in the test statistics could be observed due to deregression, PA-correction or weighting of ePOEs. With regard to the effect estimates, deregression and PA-correction did have an impact, whereas weighting had only a minor role (Fig. 2; Supplementary Table S1).

### Brown Swiss data

#### Parent-of-origin effects

**Figure 3:**
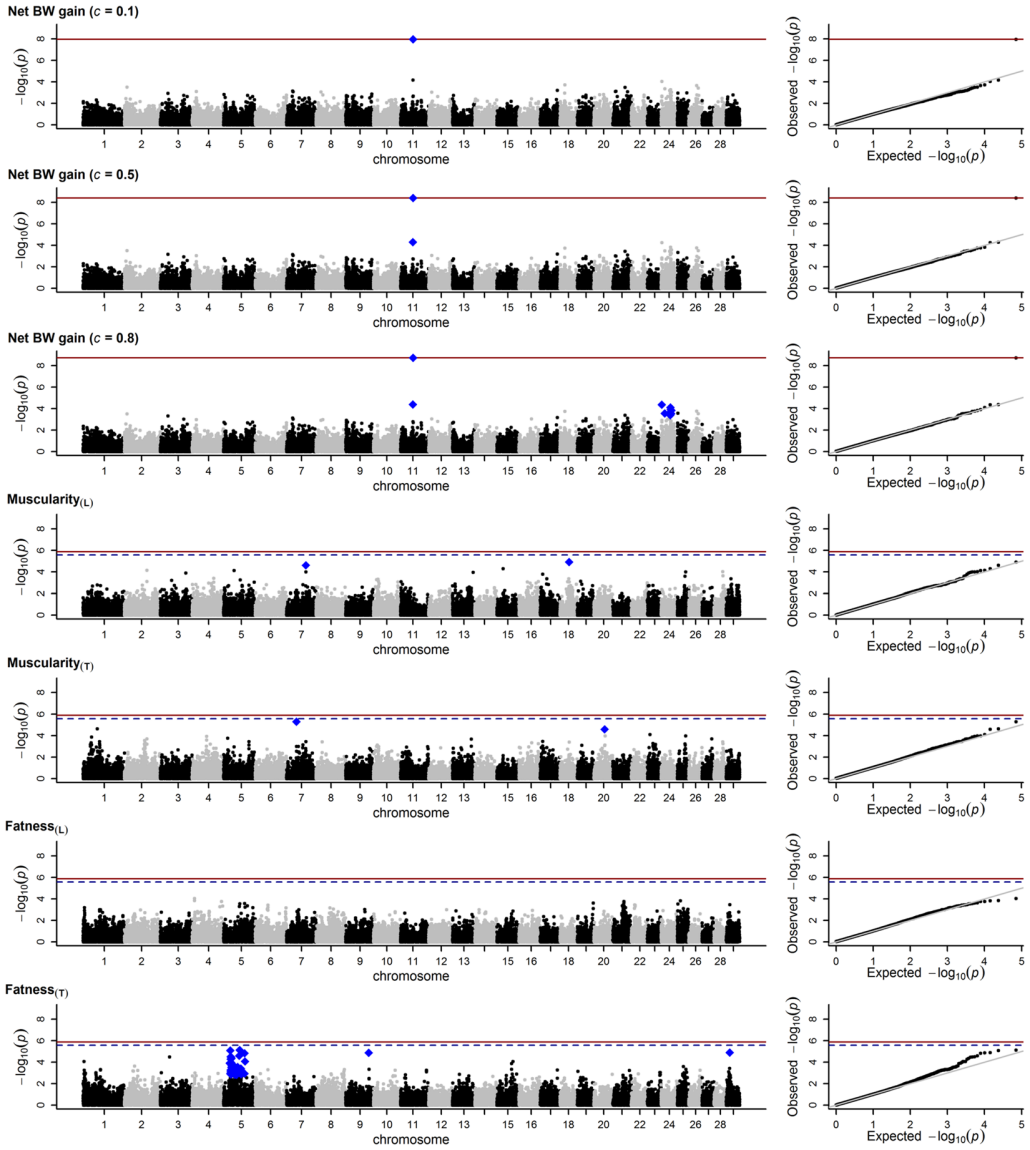
Marker loci in relation to their −log_10_ *p*-values generated by regressing estimated parent-of-origin effects (ePOEs) on marker genotypes. Net body weight (BW) gain was analysed with varying *c*-parameters of 0.1, 0.5 and 0.8. The ePOEs for carcass muscularity and carcass fatness were generated using a linear (subscript L) and threshold model (subscript T). Red line = 5% genome-wide false discovery rate (FDR); dashed line = 10% genome-wide FDR; blue diamonds = markers significant assuming a chromosome-wide FDR of 5%.

Of 37,443 single nucleotide polymorphisms (SNPs), one SNP (*ARS-BFGL-NGS-101636*) on the Bos taurus autosome (BTA) 11 was associated with ePOEs estimated in the net BW gain assuming a genome-wide false discovery rate (FDR) of 5% (Fig. 3; Table 4). As shown in Supplementary Fig. S1, SNP *ARS-BFGL-NGS-101636* can be assigned to an intron of REEP1. The imprinting status of REEP1 is unknown. This applies also to its human and mouse orthologs. Imumorin *et al*.^44^ identified bovine iQTLs in regions containing orthologs of imprinted genes in mouse and human. One bovine ortholog (HADHB) was located on BTA11 and was found in a region harbouring iQTLs with an effect on weaning weight. However, this gene is located about 24.75 mega base pairs (Mb) away from the marker locus *ARS-BFGL-NGS-101636*. Furthermore, *ARS-BFGL-NGS-101636* displayed low LD with its surrounding markers (*r*^2^ < 0.20). The highest LD (*r*^2^ = 0.34) was calculated for the SNP *BTA-97072-no-rs*, located in a non-coding region at 47.52 Mb (Supplementary Fig. S1). Thus, a causal variant may be expected within or very close to REEP1. Mutations in the human ortholog of the bovine REEP1 are related to neurodegenerative disorders such as hereditary spastic paraplegia, a syndrome characterised by progressive lower-limb spastic paralysis^45^. Furthermore, Reep1 expression has been suggested to regulate the adipogenesis in white adipose tissue in mice. In Reep1-null mice, the expression of pro-adipogenesis markers was reduced, whereas the expression of anti-adipogenesis markers was upregulated. Thereby, Reep1-null mice were observed to be thinner, albeit not lighter, than their wild-type counterparts. Males showed a significant decrease in the proportion of total adipose tissue^46^.

**Table 4:** Single nucleotide polymorphisms (SNPs) significantly associated with parent-of-origin effects (ePOEs) estimated for net body weight (BW) gain, carcass muscularity and carcass fatness. Net BW gain was analysed with varying *c*-parameters of 0.1, 0.5 and 0.8. The ePOEs for carcass muscularity and carcass fatness were generated using a linear (subscript L) and threshold model (subscript T). The estimated SNP effects are provided with standard errors (se) and *p*-values. Their base pair (bp) positions on chromosomes (chr) and known genes are indicated.

trait	SNP	effect	se	*p*-value	chr	bp	gene
Net BW gain_**0.1**_	ARS-BFGL-NGS-101636	−7.4336	1.3011	1.109e-08***	11	48473153	REEP1
Net BW gain_**0.5**_	ARS-BFGL-NGS-101636	−7.9069	1.3433	3.946e-09***	11	48473153	REEP1
	BTA-97072-no-rs	3.6728	0.9061	5.048e-05*	11	47522289	
Net BW gain_**0.8**_	ARS-BFGL-NGS-101636	−8.2353	1.3701	1.894e-09***	11	48473153	REEP1
	BTA-97072-no-rs	3.7979	0.9260	4.110e-05*	11	47522289	
	ARS-BFGL-NGS-111462	3.0791	0.7535	4.378e-05*	24	3601352	
	ARS-BFGL-NGS-37285	−3.7057	0.9391	7.951e-05*	24	36201079	
	ARS-BFGL-NGS-96321	3.5995	0.9355	1.1890-04*	24	37709361	MYOM1
	ARS-BFGL-NGS-104327	−3.5138	0.9273	1.510e-04*	24	40056388	
	BTA-05662-no-rs	3.1706	0.8647	2.463e-04*	24	36077466	
	Hapmap59495-rs29020511	−4.1534	1.1353	2.543e-04*	24	33038121	
	ARS-BFGL-NGS-31453	4.4021	1.2100	2.740e-04*	24	14704121	
	Hapmap48158-BTA-102889	−3.3790	0.9348	3.001e-04*	24	38899975	
	BTA-57995-no-rs	3.3137	0.9313	3.736e-04*	24	36003718	YES1
	BTA-57998-no-rs	3.2913	0.9311	4.091e-04*	24	36024062	YES1
Muscularity_**(L)**_	BTB-01217732	−1.1920	0.2825	2.441e-05*	7	74537346	
	Hapmap34397-BES7_Contig335_1167	−1.0794	0.2467	1.208e-05*	18	35583594	
Muscularity_**(T)**_	Hapmap41111-BTA-94756	−0.2527	0.0554	5.072e-06*	7	38413207	
	ARS-BFGL-BAC-33671	−0.1407	0.0335	2.655e-05*	20	41576197	
Fatness_**(T)**_	BTA-73718-no-rs	0.1610	0.0359	7.311e-06*	5	61790994	
	Hapmap41950-BTA-72999	−0.1663	0.0373	8.118e-06*	5	26082666	
	Hapmap39431-BTA-74343	0.2050	0.0473	1.482e-05*	5	80695234	FAR2
	ARS-BFGL-NGS-55084	−0.1464	0.0347	2.493e-05*	5	60513092	
	Hapmap39353-BTA-73120	−0.1477	0.0354	3.093e-05*	5	27777281	
	ARS-BFGL-NGS-91751	−0.1624	0.0392	3.400e-05*	5	28573792	
	ARS-BFGL-NGS-112542	0.1729	0.0423	4.332e-05*	5	30159843	FAIM2
	ARS-BFGL-NGS-113311	−0.1490	0.0368	5.210e-05*	5	27633265	KRT75
	Hapmap41784-BTA-17439	0.1663	0.0423	8.383e-05*	5	82036800	
	ARS-BFGL-NGS-7567	−0.1685	0.0433	1.025e-04*	5	28331294	SCN8A
	ARS-BFGL-NGS-77906	−0.1367	0.0356	1.208e-04*	5	24533402	TMCC3
	ARS-BFGL-NGS-10291	0.1525	0.0398	1.281e-04*	5	24512405	TMCC3
	ARS-BFGL-NGS-43909	−0.1748	0.0473	2.168e-04*	5	30879776	
	BTB-00226634	0.1250	0.0344	2.815e-04*	5	44695924	
	Hapmap42984-BTA-58358	−0.2632	0.0725	2.830e-04*	5	54449817	
	BTA-73464-no-rs	0.1294	0.0357	2.860e-04*	5	42918584	PTPRR
	ARS-BFGL-NGS-7799	0.1250	0.0345	2.906e-04*	5	44936099	
	ARS-BFGL-NGS-53461	0.1242	0.0344	3.049e-04*	5	30659497	TROAP
	ARS-USMARC-614	0.2543	0.0710	3.411e-04*	5	45722126	IL22
	ARS-BFGL-NGS-15520	0.1263	0.0356	3.878e-04*	5	65986618	GNPTAB
	ARS-BFGL-NGS-15787	−0.1319	0.0372	3.899e-04*	5	66157408	
	Hapmap47760-BTA-85576	−0.1253	0.0355	4.180e-04*	5	65998778	
	ARS-USMARC-657	0.1523	0.0435	4.653e-04*	5	45834943	IFNG
	Hapmap42150-BTA-25138	−0.1239	0.0354	4.703e-04*	5	32819286	
	ARS-BFGL-NGS-20969	0.1508	0.0435	5.207e-04*	5	62920850	
	ARS-BFGL-NGS-38038	−0.1817	0.0524	5.235e-04*	5	27992179	NR4A1
	ARS-BFGL-NGS-8796	0.1202	0.0350	5.987e-04*	5	29095603	
	ARS-BFGL-NGS-118817	−0.1885	0.0552	6.420e-04*	5	31739928	
	ARS-USMARC-652	0.1630	0.0478	6.490e-04*	5	45807839	
	ARS-USMARC-675	0.2044	0.0600	6.631e-04*	5	31164990	ADCY6
	ARS-BFGL-NGS-717	0.1202	0.0354	6.812e-04*	5	32861996	
	ARS-BFGL-NGS-30677	0.1163	0.0346	7.627e-04*	5	43565856	
	BTA-73685-no-rs	0.1234	0.0367	7.751e-04*	5	60929878	
	ARS-BFGL-NGS-7725	−0.1154	0.0344	7.835e-04*	5	27839649	KRT7
	ARS-BFGL-NGS-38020	0.1147	0.0346	9.111e-04*	5	29264678	
	ARS-BFGL-NGS-92420	0.1136	0.0344	9.721e-04*	5	33078266	
	ARS-BFGL-NGS-45045	−0.1177	0.0357	9.880e-04*	5	63899453	
	Hapmap24085-BTA-143102	−0.2532	0.0772	1.043e-04*	5	46364432	
	BTA-74351-no-rs	−0.1511	0.0466	1.168e-03*	5	80256799	TMTC1
	BTA-72978-no-rs	−0.1293	0.0398	1.175e-03*	5	25699752	NCKAP1L
	BTA-73296-no-rs	−0.1248	0.0386	1.200e-03*	5	26526934	
	ARS-BFGL-NGS-23025	0.1127	0.0351	1.338e-03*	5	66976106	PAH
	ARS-BFGL-NGS-107085	−0.1545	0.0484	1.420e-03*	5	28660813	BIN2
	BTB-01477536	−0.1139	0.0359	1.500e-03*	5	43417275	CNOT2
	ARS-BFGL-NGS-34352	0.1115	0.0351	1.507e-03*	5	49842685	SRGAP1
	ARS-BFGL-NGS-36861	−0.2174	0.0499	1.321e-05*	9	88814138	
	Hapmap33865-BES2_Contig389_1251	−0.1857	0.0426	1.280e-05*	29	13959142	

***5% genome-wide false discovery rate; *5% chromosome-wide false discovery rate.

To investigate the effect of *c* within a real data framework, we scanned the genome for ePOEs on the net BW gain, varying this parameter. With a change from *c* = 0.1 to *c* = 0.8, the estimated effects increased, whereas the *p*-values decreased (Table 4). Thus, as expected, real data with an increasing amount of heterogeneous information leads to a higher sensitivity to *c*.

In addition to *ARS-BFGL-NGS-101636*, an association significant assuming a chromosome-wide FDR of 5% was found for *BTA-97072-no-rs* nearby on BTA11 when *c* equalled 0.5. When *c* equalled 0.8, further significant markers (chromosome-wide FDR of 5%) were detected on BTA24 (Fig. 3; Table 4). Except for two SNPs, they are closely located in an area containing the genes MYOM1 and YES1. The first encodes the Myomesin-1 protein, which was found, among others, to provide a scaffold of myosin filaments^47^. The latter is involved in cytokinesis and cell cycle mechanisms^48^. The imprinting status of these genes is not known in cattle or in mice. However, with regard to the YES1 ortholog in mice, an adjacent gene (Il6) on chromosome 5 at 30.01 Mb is imprinted^49^.

With regard to muscularity_**(L)**_, two SNPs significant assuming a chromosome-wide FDR of 5% (Fig. 3; Table 4) were found in non-coding areas on BTA7 (74.53 Mb) and BTA18 (35.58 Mb). While the existence of imprinted genes on BTA7 is unknown, four maternally imprinted genes on BTA18 have been reported^49^. However, these genes are clustered in a region from 64.26 Mb to 64.54 Mb. SNPs within this cluster and the SNP found on BTA18 were independent, with *r*^2^ < 0.02. Furthermore, the significance for the SNP found on BTA18 could not be reproduced when the ePOEs estimated for muscularity_**(T)**_ were analysed. Instead, a significant SNP (chromosome-wide FDR of 5%) was found in a non-coding area on BTA7. As this marker is distantly located at 38.41 Mb, no relationship can be assumed to the SNP found on BTA7 when ePOEs on muscularity_**(L)**_ were analysed.

With regard to fatness_**(L)**_, no significant SNPs were found. However, for fatness_**(L)**_ the smallest number of records was available because 798 ePOEs were discarded from the analysis due to their low reliabilities. This might have considerably reduced the power to detect imprinted loci. With regard to fatness_**(T)**_, 45 SNPs significant assuming a 5% chromosome-wide FDR were found on BTA5. As described in detail later, a series of these SNPs were also observed to be significantly associated with the parental TAs in fatness_**(L)**_ and fatness_**(T)**_. The strongest signals were displayed by SNPs surrounding TROAP and FAIM2. These genes are located in the regions from 30.65 Mb to 30.66 Mb and 30.15 Mb to 30.18 Mb, respectively. Some SNPs, being significantly associated with ePOEs, are located within or proximal to these genes (Table 4). So far, no imprinted loci on BTA5 are known in cattle. However, the gene Slc38a4 is known to be imprinted in mice^49^. The bovine ortholog is located on BTA5 close to the genes TROAP and FAIM2 in a region from 33.61 Mb to 33.65 Mb. In a comparative expression analysis of SLC38A4 in 75 bovine tissue types (fetal and adult), Zaitoun and Khatib^50^ did not find any indication that SLC38A4 is imprinted in cattle. Another fact giving rise to some uncertainty is that the SNPs found for fatness_**(T)**_ on BTA5 were not observed when ePOEs on fatness_**(L)**_ were analysed. However, as mentioned earlier, the lowest number of records and the smallest average of reliabilities were observed for ePOEs estimated in fatness_**(L)**_. Furthermore, the heritability estimated for fatness_**(L)**_ (*h*^2^ = 0.23) in Blunk *et al*.^27^ was far below the heritability estimated for fatness_**(T)**_ (*h*^2^ = 0.46). These facts might have considerably reduced the power to detect imprinted loci when ePOEs on fatness_**(L)**_ were analysed. With regard to fatness_**(T)**_, another SNP significant assuming a 5% chromosome-wide FDR (*ARS-BFGL-NGS-36861*) was found in a non-coding region on BTA9 (Table 4). This chromosome hosts the imprinted PLAGL1 and the imprinted IGF2R. They are located in areas from 82.41 Mb to 82.47 Mb and from 97.63 Mb to 97.74 Mb, respectively. *ARS-BFGL-NGS-36861* falls in between at 88.81 Mb. The LD to SNPs proximal to PLAGL1 (*r*^2^ ≤ 0.03) and to SNPs within IGF2R (*r*^2^ ≤ 0.02), however, suggests that *ARS-BFGL-NGS-36861* is a fully independent signal. Another significant SNP (chromosome-wide FDR of 5%) was found in a non-coding region at 13.95 Mb on BTA29. An imprinting cluster is located on BTA29 incorporating the genes IGF2 and H19. However, this cluster is distantly located in the region from 49.32 Mb to 50.15 Mb.

To conclude, our results identify potentially imprinted genes on BTA5 and BTA11, affecting the carcass fatness and net BW gain, respectively. Their imprinting status cannot be established from the results of this study alone. Moreover, as ePOEs were used as dependent variables, it remains unclear whether their effects actually arose from genomic imprinting or another parent-specific genetic phenomenon (e.g. maternal genetic effects). To answer this question, follow-up studies are necessary to gain deeper insights.

#### *Transmitting abilities*

Due to strong similarities between the results for the TAs as sire and the TAs as dam, findings for the latter are subsequently omitted from Tables. The same holds true for the results found in traits analysed via the threshold model. Moreover, only markers found to be significant assuming a 5% and 10% genome-wide FDR are listed (Table 5). Detailed figures can be found in the Supplementary Material (Supplementary Fig. S4; Supplementary Table S2; Supplementary Table S3).

**Table 5:** Single nucleotide polymorphisms (SNPs) significantly associated with transmitting abilities (TAs) as sire estimated for net body weight (BW) gain, carcass muscularity and carcass fatness. The TAs for carcass muscularity and carcass fatness were generated using a linear (subscript L) and a threshold model (subscript T). The estimated SNP effects are provided with standard errors (se) and *p*-values. Their base pair (bp) positions on chromosomes (chr) and known genes are indicated.

trait	SNP	effect	se	*p*-value	chr	bp	gene
Muscularity_**(L)**_	BTB-00429961	−1.2325	0.2099	4.3065e-09***	10	56093653	UNC13C
	ARS-BFGL-NGS-21100	−1.2186	0.2098	6.2669e-09***	10	56116909	UNC13C
	BTB-01911175	1.2083	0.2100	8.7539e-09***	10	56140822	UNC13C
	BTB-00430730	1.1607	0.2118	4.2323e-08***	10	55535781	
	Hapmap51030-BTA-69263	1.2283	0.2521	1.0970e-06***	10	52190618	AQP9
	Hapmap50767-BTA-72346	1.2731	0.2852	8.0751e-06***	10	60145660	HDC
	ARS-BFGL-NGS-24556	−0.8659	0.2029	1.9888e-05**	10	51980270	
	Hapmap36252-SCAFFOLD195517_10504	−1.0007	0.2352	2.0960e-05**	10	58707718	TMOD2
	ARS-BFGL-NGS-27708	−0.8953	0.2105	2.1071e-05**	10	55510249	
	Hapmap58597-rs29013533	−0.8634	0.2049	2.5192e-05**	10	58097440	
	BTB-01125630	1.5872	0.3834	3.4723e-05**	10	57912228	
	Hapmap59786-rs29012019	−0.8747	0.2123	3.7975e-05**	10	55611885	
	ARS-USMARC-Parent-DQ984827-rs29012019	−0.8721	0.2121	3.9402e-05**	10	55611885	
	ARS-BFGL-NGS-114479	1.1821	0.2798	2.3777e-05**	16	33402551	
	ARS-BFGL-NGS-40515	−0.9244	0.2048	6.3823e-06***	23	10593430	
Fatness_**(L)**_	ARS-BFGL-NGS-67309	0.0240	0.0057	2.5727e-05***	2	114710664	
	ARS-BFGL-NGS-38020	0.0328	0.0052	2.9461e-10***	5	29264678	
	Hapmap39353-BTA-73120	−0.0335	0.0055	7.9597e-10***	5	27777281	
	ARS-BFGL-NGS-53461	0.0292	0.0052	1.7002e-08***	5	30659497	TROAP
	ARS-BFGL-NGS-91751	−0.0332	0.0059	1.9337e-08***	5	28573792	
	ARS-BFGL-NGS-92420	0.0293	0.0053	2.5670e-08***	5	33078266	
	ARS-BFGL-NGS-30168	0.0266	0.0054	8.8196e-07***	5	111313740	SYNGR1
	ARS-BFGL-NGS-98156	0.0255	0.0053	1.5540e-06***	5	27869236	
	Hapmap42150-BTA-25138	−0.0257	0.0054	2.2840e-06***	5	32819286	
	ARS-USMARC-675	0.0410	0.0087	2.5085e-06***	5	31164990	ADCY6
	Hapmap51043-BTA-73218	0.0230	0.0049	2.8574e-06***	5	29622395	LARP4
	ARS-BFGL-NGS-107085	−0.0330	0.0071	2.9176e-06***	5	28660813	BIN2
	ARS-BFGL-NGS-112542	0.0288	0.0063	4.0947e-06***	5	30159843	FAIM2
	BTA-72978-no-rs	−0.0274	0.0060	4.2470e-06***	5	25699752	NCKAP1L
	Hapmap23022-BTA-161235	−0.0245	0.0053	4.2916e-06***	5	31613026	
	ARS-USMARC-629	0.0310	0.0068	5.6301e-06***	5	25738874	GTSF1
	ARS-USMARC-657	0.0281	0.0062	5.8398e-06***	5	45834943	IFNG
	BTA-73209-no-rs	0.0230	0.0051	6.6548e-06***	5	29496625	DIP2B
	ARS-BFGL-NGS-68582	−0.0251	0.0056	7.7038e-06***	5	111380409	
	ARS-BFGL-NGS-717	0.0240	0.0055	1.0656e-05***	5	32861996	
	ARS-BFGL-NGS-7725	−0.0220	0.0051	1.3210e-05***	5	27839649	KRT7
	BTA-73392-no-rs	0.0243	0.0056	1.4981e-05***	5	32104484	
	ARS-BFGL-NGS-8796	0.0227	0.0054	2.9035e-05***	5	29095603	
	ARS-USMARC-652	0.0282	0.0069	4.0453e-05**	5	45807839	
	BTA-28787-no-rs	0.0221	0.0054	4.4479e-05**	5	37925443	
	ARS-BFGL-NGS-11851	0.0226	0.0055	4.5188e-05**	5	32426908	
	UA-IFASA-2781	0.0218	0.0054	5.3496e-05**	5	29989860	ASIC1
	ARS-BFGL-NGS-43909	−0.0282	0.0070	5.4350e-05**	5	30879776	
	Hapmap55179-rs29024483	0.0231	0.0055	2.9496e-05***	13	38830375	DZANK1
	Hapmap55095-rs29010810	0.0260	0.0065	7.0403e-05**	13	27589301	
	ARS-BFGL-NGS-4866	−0.0317	0.0079	5.3215e-05**	16	50095343	
	ARS-BFGL-NGS-18515	0.0231	0.0055	2.2798e-05***	19	29219211	NTN1

***5% genome-wide false discovery rate; **10% genome-wide false discovery rate.

**Figure 4:**
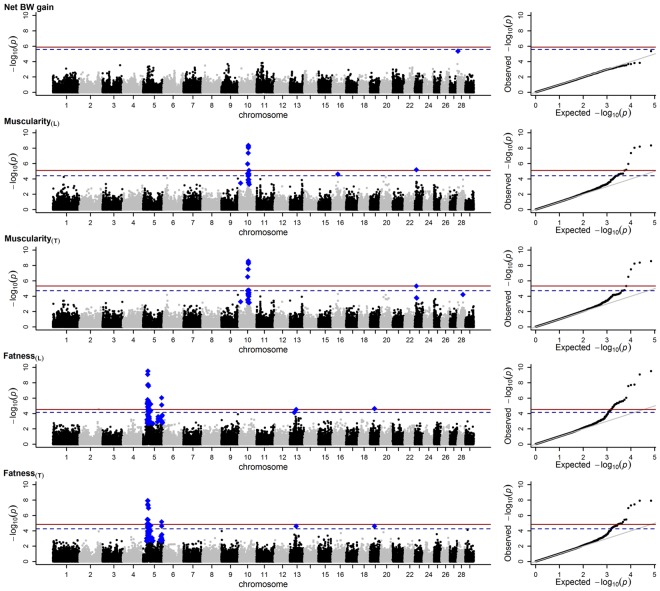
Marker loci in relation to their −log_10_ *p*-values generated by regressing transmitting abilities (TAs) as sire on marker genotypes. The TAs for carcass muscularity and carcass fatness were generated using a linear (subscript L) and threshold model (subscript T). Red line = 5% genome-wide false discovery rate (FDR); dashed line = 10% genome-wide FDR; blue diamonds = markers significant assuming a chromosome-wide FDR of 5%.

With regard to net BW gain, only one SNP, located in a non-coding region on BTA28, was found to be significantly associated with the parental TAs assuming a chromosome-wide FDR of 5% (Fig. 4; Supplementary Fig. S4; Supplementary Table S2; Supplementary Table S3).

With regard to muscularity_**(L)**_, six SNPs on BTA10 were associated with the parental TAs assuming a 5% genome-wide FDR (Fig. 4; Table 5). Except for one SNP, these findings were reproduced for both parental TAs estimated in muscularity_**(T)**_. Three SNPs can be assigned to the gene UNC13C and one SNP to the gene AQP9. Their effects are of similar magnitude (Table 5), and slightly higher effects were observed when the TAs as dam were analysed (Supplementary Table S3). This holds true for muscularity_**(L)**_ as well as for muscularity_**(T)**_. Both genes are protein coding; however, their functions are still unclear in cattle. They are located in the regions from 52.15 Mb to 52.19 Mb (AQP9) and from 55.71 Mb to 56.41 Mb (UNC13C). Moderate lambdas (up to 1.042 for the TAs as sire) indicate almost no genome-wide inflation of *p*-values^51^, as shown for the muscularity traits in the Q-Q plot in Fig. 4. However, SNPs located in UNC13C and AQP9 are not fully independent, with *r*^2^ > 0.24 (for LD patterns and gene assignments on BTA10 see Supplementary Fig. S3). Therefore, whether causal variants are located in either UNC13C, AQP9 or in both cannot clearly be determined. According to the CattleQTLdb (Release 32^52^), a QTL associated with lean meat yield was found in Holstein-Friesian on BTA10 at 56.10 Mb, a region which is located within UNC13C^53^. Apart from the findings on BTA10, further markers significantly associated with the TAs estimated in the muscularity traits were found on BTA20, BTA23 and BTA28. Associations on BTA20 were only found for the TAs as dam assuming a chromosome-wide FDR of 5%. They cannot be assigned to genes. However, Doran *et al*.^53^ detected SNPs on BTA20, which were strongly associated with carcass conformation and were <1 Mb away from the gene GHR. In the current study, one of the SNPs on BTA20 is located only 41.75 Kb away from GHR.

A SNP on BTA23 was found to be significantly associated with both parental TAs estimated in muscularity_**(L)**_ and muscularity_**(T)**_ with a 5% genome-wide FDR. However, it is located within a non-coding region, and markers found to be significantly associated with carcass conformation in Doran *et al*.^53^ are distantly located. Another marker significantly associated (chromosome-wide FDR of 5%) with both TAs estimated in the muscularity_**(T)**_ was found on BTA28 within the gene KCNMA1 (32.82 Mb to 33.58 Mb). Its function with regard to the carcass conformation in cattle is unknown. However, Doran *et al*.^53^ identified a QTL with an effect on lean meat yield close to this gene at 33.60 Mb (CattleQTLdb; Release 32^52^).

With respect to fatness_**(L)**_, 22 SNPs on BTA5 were found to be associated with the TAs as sire assuming a genome-wide FDR of 5%. The smallest *p*-values (*p* < 2.6e-08) were displayed by markers surrounding TROAP located in a region from 30.65 Mb to 30.66 Mb. Overall, 41 further SNPs on BTA5 were significant assuming a genome-wide FDR of 10% or a chromosome-wide FDR of 5%. They were replicated for the TAs as dam as well as for the parental TAs estimated in fatness_**(T)**_. Some SNPs are located within genes other than TROAP (Table 5). However, due to their moderate to high LD to SNPs located within TROAP, most of them may not be causal but indicators for causal variants within or proximal to TROAP (Supplementary Fig. S2). According to the CattleQTLdb (Release 32^52^), haplotypes with an effect on backfat breeding values are closely located to TROAP in a region from 32.6 Mb to 34.2 Mb^54^. Further significant SNPs (genome-wide FDR of 5% and 10%) were found on BTA2, BTA13, BTA16 and BTA19 when the TAs as sire estimated in fatness_**(L)**_ were used as dependent variables (Fig. 4; Table 5). The SNP on BTA13 is located in the gene DZANK1, which is positioned in an area from 38.78 Mb to 38.83 Mb. McClure *et al*.^55^ found a QTL with an effect on fat thickness within this area (CattleQTLdb; Release 32^52^). With regard to SNPs solely found when TAs as dam were analysed, a SNP significant assuming a 5% chromosome-wide FDR was revealed for fatness_**(T)**_ on BTA29 (Supplementary Fig. S4; Supplementary Table S3). The SNP (13.59 Mb) is located far from IGF2 (50.04 Mb to 50.06 Mb). Therefore, a connection seems unlikely.

To conclude, the strongest indications for markers associated with TAs observed in the muscularity and fatness traits were found on BTA10 and BTA5, respectively. These signals mainly pointed to variants located within or proximal to the genes UNC13C (BTA10) and TROAP (BTA5). Scanning the genome for effects on TAs, which were estimated in the muscularity and fatness traits via linear and threshold models, led to results of high consistency. This suggests — at least for the categorical traits under consideration — that the linear and threshold models perform equally well at capturing the underlying genetic variation.

## General Discussion

From the single-locus population model with imprinting it was derived that the regression of true POEs on allele counts equals the imprinting effect. This was the foundation for our simulation study, by which estimates of POEs were related to marker alleles. In a two-step procedure, ePOEs and their reliabilities were first obtained by employing appropriate mixed models. In this way, information on parent-of-origin-specific genetic effects was extracted from un-genotyped offspring and summarised for genotyped parents. In a second step, the deregressed ePOEs then served as dependent variables to be regressed on allele counts of marker alleles, indicating the association of an iQTL with the marker. Most importantly, in this way, the complete lack of any genotype information for progeny and the phenotypes of the parents could be overcome. This is a substantial step forward compared to previously published methods for association mapping of imprinted loci, as these require data where phenotypes and ordered genotypes are known for the same individuals (GRAMMAR^32,33^; measured-genotype approach^43^). Available marker information could, in principle, already be integrated in the estimation of ePOEs by replacing the numerator relationship matrix in the mixed model through a combined marker and pedigree-based relationship matrix ***H***^56^. Following Wang *et al*.^57^, ePOEs of genotyped parents could then be transformed into marker effects and potential QTL-regions be made visible. However, significance testing of such marker effects has, to our best knowledge, not yet been described in the literature.

The Brown Swiss beef traits data set is a meat animal example, where phenotype-providing progeny never become parents and remain un-genotyped. In livestock genetics, other candidate data show the same characteristics, e.g. in dual-purpose Simmental^28^ or Gelbvieh cattle^58^. In other cases, however, accumulating data from ongoing breeding programmes may often also comprise a certain proportion of phenotyped progeny that also has ordered genotypes available (e.g. Guo *et al*.^59^) and, to a certain fraction, may also be parents themselves (e.g. Jiang *et al*.^60^). This calls for analyses of mixed data, where the vector of dependent variables contains ePOEs for genotyped parents, as a summary of information from un-genotyped progeny, and the phenotypes of genotyped progeny. Their respective counterparts on the explanatory side of the model are marker allele counts and ordered genotype information. Thus, including ePOEs in marker-based imprinting analyses enables researchers to make full use of available information which otherwise would remain un-accessible.

How power and cost effectiveness of the measured-genotype approach^43^ (with genotypes on progeny only) and our new approach compare depends on several factors. For the use of ePOEs sufficient data of adequate structure (sires and dams have to be related) are required to estimate variance components. Another important point is the average reliability of ePOEs. With genotyped progeny we also need to assign resources to the genotyping of their parents, otherwise we cannot derive the parental origin of marker alleles. In general, our new approach is obviously well suited for further studies of the importance of POEs in species where large progeny groups can be produced or are available and their phenotypic information can be combined into reliable estimates of parental POEs. This is especially the case in plants and some animal species with large half-sib families, such as horses, cattle or birds.

Some authors have stressed the fact that POEs may be caused by other phenomena than imprinting alone, e.g. by maternal genetic effects^61^. Accordingly, ePOEs may pick up such effects, especially when not explicitly accounted for in the model for POE-estimation (e.g. Blunk *et al*.^27,28^). Other potential confounders are Y-chromosomal and mitochondrial effects, which, however, seem to be negligible at least for beef traits^24,25,62^. However, if present, the latter would not show any signal of association with purely autosomal markers, as investigated in the Brown Swiss analysis. Moreover, model-specification is also important for the ability to find associations between different kinds of imprinted loci and markers. Models that account for all kinds of genomic imprinting simultaneously, no matter if paternal, maternal, fully or partial, have not been described in the literature before^24,25^. When such models are used for POE-estimation, all types of imprinted loci can, in principle, be located in the genome, as was successfully demonstrated in the simulation study. Nevertheless, follow-up studies are necessary, as for other approaches like mapping in line-cross experiments^39^, in order to confirm both genomic imprinting and its exact type.

As shown for breeding values in Ekine *et al*.^42^, preprocessing the ePOEs is necessary to achieve reliable marker effect estimates and to avoid large false-positive rates. With regard to the weighting, *c* was limited to 0.1 to analyse the muscularity and fatness traits. When the ePOEs on net BW gain were analysed, *c* was varied, which led to some changes in the *p*-values and estimated effects. As this indicates a degree of sensitivity to *c*, varying this parameter with regard to the muscularity and fatness traits might have resulted in the detection of additional associations. However, as no effect of the weighting was observed in the simulation study, it remains questionable whether changing *c* actually results in true associations or in an inflation of effects. Thus, whether the associations found on BTA24 for ePOEs on the net BW gain constitute true associations or false-positives remained unclear when *c* was set to 0.8. Analyses with greater amounts of data would help investigating the true imprinting status of loci, especially regarding the SNPs found on BTA24.

Note that the approximate method of deregression and PA-correction according to Garrick *et al*.^41^ did not allow the deregression of two genetic effects simultaneously, which would, however, be appropriate using models that consider imprinting by including two genetic effects. In an article on the international genetic evaluation of beef cattle weaning weight^63^, the separate deregression of direct genetic effects and maternal genetic effects was described. A correction was presented, in which the contributions of the second correlated effect to the variance of the first are eliminated, when the first genetic effect is deregressed. With the help of a λ-value, corrected heritability was computed both for the direct and maternal effect, which can be interpreted as equivalent heritability under a single trait model^63^. The same principle could be applied to the reduced *imprinting model* (for details see the Supplementary Material). However, using an adapted λ-value in the approximation method by Garrick *et al*.^41^ did not have any impact as the λ-value was cancelled out (proof is provided in the Supplementary Material).

In summary, the possibility to detect imprinted loci by regressing ePOEs of parents on the gene counts of their un-ordered marker genotypes has not been recognised before. Fields of application are, in principle, all kinds of pedigreed populations, be it animals or plants, where genotyping of parents has become part of routine breeding procedures, or, possibly even in human populations with available detailed genealogies^64^. Apart from whole-genome scans, targeted analyses may also concentrate on associations in and around known imprinted genes. Further research will aim at more general analyses that include phenotypes from individuals also providing ordered genotype information. In any case, vast repositories of already collected phenotypes will now become accessible for imprinting analyses by applying our new approach.

## Methods

### Simulated data

To estimate the ePOEs (needed for the analyses 1A, 3A, 3B, and 3C) for each parent together with their respective reliabilities, the phenotypic information from the progeny in the last generations were summarised by applying a reduced *imprinting model* (as described in Blunk *et al*.^27^). Estimates for the TAs (needed for analysis 1B) were derived from a reduced animal model that assumes no imprinting (as described in Neugebauer *et al*.^24,25^). Then, the model:

$${y}_{i}=\mu +b{x}_{i}^{a}+{u}_{i}+{e}_{i},$$

was applied consecutively for each marker. The dependent variable *y*_*i*_ was either the ePOE (analyses 1A, 3A, 3B, and 3C) or the TA of individual *i* (analysis 1B), *μ* was the general mean and *e*_*i*_ represents the residual. The term ${x}_{i}^{a}$ is the gene content (0, 1 or 2 for the respective known marker genotypes *BB*, *Bb* and *bb*) of individual *i* at the particular marker under consideration and *b* is the regression coefficient. A significant test result of the hypothesis H_0_: *b* = 0 indicates the association of a marker with an iQTL in case the dependent variable is an ePOE (analysis 1A) and the association of a marker with an additive QTL in case *y*_*i*_ is a TA (analysis 1B). Tests were done separately for each marker via a conditional Wald *F*-test using the ASReml-package (Release 3.0^65^) and ASReml-R (Version 3^66^). The random variable *u*_*i*_ models the unaccounted genetic variability and was assumed to have a variance of *Var*(***u***) = ***Aσ***^2^ with ***A*** as the numerator relationship matrix (derived from the pedigree), which was included to account for the stratification of the population into families. Note, in the analyses 1A and 1B, *u*_*i*_ was not included because the individuals with genotypes were unrelated founders. A summary of analyses is provided in Table 2. To find associations with imprinted loci, in analysis 2A an adapted version of a measured genotype model^43^:

$${y}_{i}=\mu +{b}_{a}{x}_{i}^{a}+{b}_{p}{x}_{i}^{p}+{g}_{i}^{s}+{g}_{i}^{d}+{e}_{i},$$

was applied consecutively to each marker. Here *y*_*i*_ is the observed phenotype. The regression coefficient *b*_*a*_ can be interpreted as the additive effect of the locus. The regression coefficient *b*_*p*_ delivers an estimate for the difference between the two types of heterozygotes *Bb* and *bB*, i.e. for the imprinting effect. The term ${x}_{i}^{p}$ has the values of 0, 1, −1, and 0 for the ordered genotypes *BB*, *Bb*, *bB* and *bb*, respectively. The association with an iQTL is equivalent to a test of H_0_: *b*_*p*_ = 0. It was assumed that the random gametic effects ${g}_{i}^{s}$ (effect of the father’s gamete) and ${g}_{i}^{d}$ (effect of the mother’s gamete) had different variances ${\sigma }_{s}^{2}$ and ${\sigma }_{d}^{2}$ from a multivariate normal distribution, with the variance:

$$Var[\begin{array}{c}{{\boldsymbol{g}}}^{{\boldsymbol{s}}}\\ {{\boldsymbol{g}}}^{{\boldsymbol{d}}}\end{array}]={\boldsymbol{G}}\otimes [\begin{array}{cc}{\sigma }_{s}^{2} & {\sigma }_{{\rm{sd}}}\\ {\sigma }_{{\rm{sd}}} & {\sigma }_{d}^{2}\end{array}],$$

with ***G*** as the gametic relationship matrix^67,68^. These random gametic effects were included to account for the stratification of the population into families. Analysis 2B considered additive effects only by neglecting ${b}_{p}{x}_{i}^{p}$. A test for H_0_: *b*_*a*_ = 0 was performed for each marker separately.

With regard to the analyses 3A, 3B, and 3C, the deregression, PA-correction and weighting of the ePOEs were achieved by adapting the approximate method published in Garrick *et al*.^41^ for breeding values, which is described in detail in the Supplementary Material for ePOEs. To define parameter *c*, which is needed to calculate the weightings, a grid search was conducted, where *c* was progressively increased with a step size of 0.05 according to a proposal in Gorjanc *et al*.^69^. The *c*-parameter generating the greatest log-likelihood was chosen for each considered marker.

### **Brown Swiss data**

The ePOEs and parental TAs as well as their reliabilities were derived from an imprinting analysis of Brown Swiss cattle slaughterhouse data published in detail in Blunk *et al*.^27^. The reduced *imprinting model* was used to estimate the ePOEs as well as the TAs.

Genotype information was acquired from two versions (v1 and v2) of the BovineSNP50 BeadChip (Illumina, San Diego, CA, USA). Thirty SNPs map to the same position (base pairs). Note that they were kept for the analyses. Using PLINK version v1.07^70^, genotypes were excluded based on low frequencies (MAF < 0.05) and a Hardy-Weinberg equilibrium test (*p* ≤ 10^−5^). Neither animals nor genotypes were dismissed due to bad genotyping quality as missing genotypes were imputed beforehand (BEAGLE version 3.3.2^71^).

The genome was scanned via single marker regression, where ePOEs (defined as deviations of the TAs as dam from the TAs as sire), the TAs as sire and the TAs as dam were regressed on their marker gene counts individually. The marker effect’s deviation from zero was tested by conducting a conditional Wald *F*-test using ASReml (Release 3.0^65^). To control the type I error rate, the significance threshold of *p*-values was adjusted according to the genome-wide FDR^72^. Weaker signals only exceeding a chromosome-wide FDR of 5% were additionally reported. The unexplained genetic variance due to the relationships between sires was captured by including the inverse of the additive genomic relationship matrix^73^. The matrix was constructed with all available markers, i.e. the marker tested was not excluded from the matrix^74^. As it was not of full rank, its blending^73^ with 5% of the numerator relationship matrix ***A*** was required. Matrix ***A*** only contained the relationships between the animals with genotypes and was generated using the kinship2 R-package version 1.6.4^75^ in R^76^.

Except otherwise specified, all positions of SNPs and further genetic information were obtained from the UCSC Genome Browser (http://genome.ucsc.edu/) based on the Bos Taurus UMD3.1.1/bosTau8 (assembly date: Dec. 2009). Pairwise LD between SNPs was specified as *r*^2^ estimated using Haploview (version 4.2^77^). Information about the known imprinting status of genes was derived from the geneimprint database^49^.

## Supplementary information

Supplementary Information

## Data Availability

Simulated data. The code used to generate the simulated data is freely available from the corresponding author upon request. The simulation results as well as corresponding programmes to visualise these results (*F*-values, *p*-values, estimated marker effects and standard errors for each marker and replication) are available at: https://doi.org/10.22000/81. Brown Swiss data. The Brown Swiss data merely served as an application example for our new method. The datasets are not publicly accessible as they were made available on a confidential basis due to commercial sensivity. The data would be available from our co-author Henning Hamman upon reasonable request but restrictions apply to the availability of these data (Material Transfer Agreement). Programmes to visualise the results as well as locations of SNPs and their *p*-values are, however, available for all traits at the above mentioned link.
