# Supplementary material for: Scanning the genomes of parents for imprinted loci acting in their un-genotyped progeny
Source: Sci Rep. 2019 Jan 24;9:654. doi: 10.1038/s41598-018-36939-3 (PMC6345920; doi:10.1038/s41598-018-36939-3)
Supplement: Supplementary file 1 — Supplementary Information [file 41598_2018_36939_MOESM1_ESM.pdf]

Title: Scanning the genomes of parents for imprinted loci acting in their un-genotyped progeny

Authors: Inga Blunk[1], Manfred Mayer[2], Henning Hamann[3], Norbert Reinsch[2]*

Author affiliations:

[1]Faculty of Agricultural and Environmental Sciences, University of Rostock,
Justus-von-Liebig-Weg 6, 18059 Rostock, Germany.

[2]Institute of Genetics and Biometry, Leibniz Institute for Farm Animal Biology (FBN),
Wilhelm-Stahl-Allee 2, 18196 Dummerstorf, Germany.

[3]State-Office for Geo-Information and Rural Development, Geodata-Center,
Stuttgarter Straße 161, 70806 Kornwestheim, Germany.

*Norbert Reinsch

Leibniz Institute for Farm Animal Biology (FBN)

Institute of Genetics and Biometry

Wilhelm-Stahl-Allee 2

18196 Dummerstorf, Germany

Phone: +49 38208 68 900

E-mail: reinsch@fbn-dummerstorf.de

Supplementary Notes

Deregression, parent-average correction and weighting

The reliability of an estimated parent-of-origin effect (ePOE) of an individual *j* was determined with $r_j^2 = 1 - PEV \Big/ \left(\tfrac{1}{2}\sigma_i^2\left(1+F_j\right)\right)$, where the *PEV* is the prediction error variance and F_j is the inbreeding coefficient (generated using the pedigree R-package version 1.4[1] in R[2]). To compute the parent average (PA) corrected ePOEs analogous to Garrick *et al.*[3], the PA reliabilities (r_{PA}^2) and the PA ePOEs ($\hat{i}_{PA}$) were necessary. Moreover, a lambda was needed, which in our case corresponds to $\lambda = \sigma_R^2 / \tfrac{1}{2}\sigma_i^2$, where σ_R^2 is the residual variance obtained from the reduced *imprinting model*[4] and σ_i^2 is the estimated imprinting variance. The inverse covariance matrix for the PA and descendants assuming uncorrelated sire and dam effects is $\boldsymbol{A}^{-1} = \begin{bmatrix} 4 & -2 \\ -2 & 2 \end{bmatrix}\sigma_i^{-2}$.

Then, the following equations were set up:

$$\begin{bmatrix} Z_{PA}'Z_{PA} + 4\lambda & -2\lambda \\ -2\lambda & Z_j'Z_j + 2\lambda \end{bmatrix}\begin{bmatrix} \hat{i}_{PA} \\ \hat{i}_j \end{bmatrix} = \begin{bmatrix} y_{PA}^* \\ y_j^* \end{bmatrix}, \tag{1}$$

where $\hat{i}_j$ corresponds to the ePOE of individual *j*. According to Garrick *et al.*[3], the direct solution for $Z_j'Z_j$ is $Z_j'Z_j = \delta Z_{PA}'Z_{PA} + 2\lambda\left(2\delta - 1\right)$ with $\delta = \left(0.5 - r_{PA}^2\right) / \left(1 - r_j^2\right)$. The direct solution for $Z_{PA}'Z_{PA}$ is $Z_{PA}'Z_{PA} = \lambda\left(0.5\alpha - 4\right) + 0.5\lambda\sqrt{\left(\alpha^2 + 16/\delta\right)}$ with $\alpha = 1 \Big/ \left(0.5 - r_{PA}^2\right)$. The left-hand-side of equation (1) was then reconstructed and y_{PA}^* and y_j^* were calculated. The equation $\left[Z_j'Z_j + \lambda\right]\left[\hat{i}_{j\text{-}PA}\right] = \left[y_j^*\right]$ was solved for the PA-corrected ePOE $\hat{i}_{j\text{-}PA}$ and the corresponding PA-corrected reliability r_j^{2*} was achieved as $r_j^{2*} = 1 - \lambda \Big/ \left(Z_j'Z_j + \lambda\right)$. Following the PA-correction, the $\hat{i}_{j\text{-}PA}$ were deregressed, which involved their division by r_j^{2*}. In Garrick *et al.*[3],

this is simplified by $y_j^* / Z_j^{'} Z_j$. Finally, each deregressed ePOE needed to be weighted. The polygenic part of the ePOE not explained by markers is $var\left(\varepsilon_j\right)$. Adding the deregressed prediction error variance, the residual variance of a deregressed ePOE by applying the principles that were outlined in Garrick *et al.*[3] for deregressed breeding values is:

$$var\left(\varepsilon_j + k_j \hat{i}_j - i_j\right) = var\left(\varepsilon_j\right) + \frac{\left(1 - r_j^{2*}\right)}{r_j^{2*}} var\left(i_j\right) = \sigma_\varepsilon^2 + \frac{\left(1 - r_j^{2*}\right)}{r_j^{2*}} \sigma_i^2 = \left[c + \frac{\left(1 - r_j^{2*}\right)}{r_j^{2*}}\right] \sigma_i^2 .$$

The parameter *c* defines the proportion of σ_i^2 not captured by markers, so that $var\left(\varepsilon_j\right) = c\sigma_i^2$.

To define *c*, a grid search was conducted, where *c* was progressively increased with a step size of 0.05 according to a proposal in Gorjanc *et al.*[5]. The *c* generating the greatest log-likelihood was chosen for each considered marker. Eventually, the inverse weights can be written as:

$$w_j^{-1} = \frac{\sigma_R^2}{\left[c + \left(1 - r_j^{2*}\right) / r_j^{2*}\right] \sigma_i^2} .$$

As explained earlier, the approximate method of deregression and PA-correction according to Garrick *et al.*[3] assumes a single genetic effect in the estimation model and a corresponding λ-value. With two genetic effects, a simultaneous deregression of both could be seen as appropriate. However, in an article on the international genetic evaluation of beef cattle weaning weight[6], the separate deregression of two genetic effects from the same model was described. A correction was presented, where contributions of the second correlated effect to the variance of the first are eliminated, when the first genetic effect is deregressed. With the help of that λ-value, a corrected heritability can be computed for both effects, which can be interpreted as an equivalent heritability under a single trait model[6]. The same principle can be applied to ePOEs from the reduced *imprinting model*[4], where λ can be written as:

$$\lambda = \frac{\frac{1}{2}\sigma_s^2 + \frac{1}{2}\sigma_d^2 + \sigma_e^2}{\frac{1}{2}\sigma_s^2} = \frac{\sigma_R^2}{\frac{1}{2}\sigma_s^2} = \frac{\frac{1}{2}\sigma_s^2 + \sigma_e^2}{\frac{1}{2}\sigma_s^2} + \frac{\frac{1}{2}\sigma_d^2}{\frac{1}{2}\sigma_s^2},$$

where σ_s^2 is the paternal gametic variance, σ_d^2 is the maternal gametic variance and σ_e^2 is the residual variance. The λ-value can then be adjusted by subtracting $\frac{1}{2}\sigma_d^2 \big/ \frac{1}{2}\sigma_s^2$, i.e.:

$$\lambda^* = \lambda - \frac{\frac{1}{2}\sigma_d^2}{\frac{1}{2}\sigma_s^2} = \frac{\frac{1}{2}\sigma_s^2 + \sigma_e^2}{\frac{1}{2}\sigma_s^2}.$$

Through this adjustment to a hypothetical single trait model, λ* becomes smaller than λ, indicating a higher contribution of $\frac{1}{2}\sigma_s^2$ to the total phenotypic variation in the hypothetical single trait model.

For the POEs, we express $\frac{1}{2}\sigma_d^2$ in terms of $\sigma_i^2 = \sigma_s^2 + \sigma_d^2 - 2\sigma_{sd}$, which is then $\frac{1}{2}\sigma_d^2 = \frac{1}{2}\sigma_i^2 - \frac{1}{2}\sigma_s^2 + \sigma_{sd}$. Then $\sigma_R^2 = \frac{1}{2}\sigma_s^2 + \frac{1}{2}\sigma_i^2 - \frac{1}{2}\sigma_s^2 + \sigma_{sd} + \sigma_e^2 = \frac{1}{2}\sigma_i^2 + \sigma_{sd} + \sigma_e^2$ and $\lambda = \frac{\frac{1}{2}\sigma_i^2 + \sigma_{sd} + \sigma_e^2}{\frac{1}{2}\sigma_i^2} = \frac{\frac{1}{2}\sigma_i^2 + \sigma_e^2}{\frac{1}{2}\sigma_i^2} + \frac{\sigma_{sd}}{\frac{1}{2}\sigma_i^2}$ so that $\lambda^* = \lambda - \frac{\sigma_{sd}}{\frac{1}{2}\sigma_i^2} = \frac{\frac{1}{2}\sigma_i^2 + \sigma_e^2}{\frac{1}{2}\sigma_i^2}$. Again λ* can be used in a separate deregression of POEs as if the original estimates have been obtained from a single-trait model again. However, using λ* in the approximation method by Garrick *et al.*[3] did not have any impact, as λ* cancels out. This can be demonstrated by the following explanations. As shown earlier, $Z'_{PA}Z_{PA} = \lambda\left(0.5\alpha - 4\right) + 0.5\lambda\sqrt{\left(\alpha^2 + 16/\delta\right)}$. This can be also written as $Z'_{PA}Z_{PA} = \lambda\left[\left(0.5\alpha - 4\right) + 0.5\sqrt{\left(\alpha^2 + 16/\delta\right)}\right]$. This expression can be used to calculate Z'_jZ_j with $Z'_jZ_j = \delta\lambda\left[(0.5\alpha - 4) + 0.5\sqrt{(\alpha^2 + 16/\delta)}\right] + 2\lambda(2\delta - 1)$, or $Z'_jZ_j = \lambda\left[\delta(0.5\alpha - 4) + 0.5\delta\sqrt{(\alpha^2 + 16/\delta)} + 2(2\delta - 1)\right]$. According to equation (1), $y_j^* = -2\lambda\hat{i}_{PA} + \left(Z'_jZ_j + 2\lambda\right)\hat{i}_j$ so that $y_j^* \big/ Z'_j Z_j$ (the parent average corrected and deregressed

ePOEs) can be expressed as $\frac{y_j^*}{Z_j^{'}Z_j} = \frac{-2\lambda \hat{i}_{PA} + \left(Z_j^{'}Z_j + 2\lambda \right) \hat{i}_j}{Z_j^{'}Z_j}$ or, equivalently,

$$\frac{y_j^*}{Z_j^{'}Z_j} = \frac{-2\lambda \hat{i}_{PA} + \left(\lambda \left[\delta(0.5\alpha - 4) + 0.5\delta\sqrt{(\alpha^2 + 16/\delta)} + 2(2\delta - 1) \right] + 2\lambda \right) \hat{i}_j}{\lambda \left[\delta(0.5\alpha - 4) + 0.5\delta\sqrt{(\alpha^2 + 16/\delta)} + 2(2\delta - 1) \right]}$$, where λ cancels out: $$\frac{y_j^*}{Z_j^{'}Z_j} = \frac{-2\hat{i}_{PA} + \left(\left[\delta(0.5\alpha - 4) + 0.5\delta\sqrt{(\alpha^2 + 16/\delta)} + 2(2\delta - 1) \right] + 2 \right) \hat{i}_j}{\left[\delta(0.5\alpha - 4) + 0.5\delta\sqrt{(\alpha^2 + 16/\delta)} + 2(2\delta - 1) \right]}.$$

In conclusion, the approximation method of Garrick *et al.*[3] is insensitive to λ or λ^*. When other methods of deregression are employed, which were originally tailored for single-trait models[6], the appropriate parameter λ^* should, of course, be chosen.

References

1. Coster, A. *Pedigree: pedigree functions. R package version 1.4* (2013). http://cran.r-project.org/web/packages/pedigree/index.html [last accessed in July 2017].
2. R Core Team, R: *A language and environment for statistical computing*. R Foundation for Statistical Computing, Vienna, Austria (2015). http://www.R-project.org/ [last accessed in July 2017].
3. Garrick, D. J., Taylor, J. F. & Fernando, R. L. Deregressing estimated breeding values and weighting information for genomic regression analyses. *Genet. Sel. Evol.* **41,** 55; 10.1186/1297-9686-41-55 (2009).
4. Blunk, I., Mayer, M., Hamann, H. & Reinsch, N. A new model for parent-of-origin effect analyses applied to Brown Swiss cattle slaughterhouse data. *Animal* **11,** 1096–1106 (2017).

5. Gorjanc, G., Woolliams, J. A. & Hickey, J. M. Hierarchical quantitative genetic model using genomic information. *Proc. 10th World Congr. Genet. Appl. Livest. Prod.*, 17–22 August 2014, Vancouver, BC, Canada, **068** (2014).
6. Phocas, F., Donoghue, K. & Graser, H. U. Investigation of three strategies for an international genetic evaluation of beef cattle weaning weight. *Genet. Sel. Evol.* **37,** 361–380 (2005).

Supplementary Figures

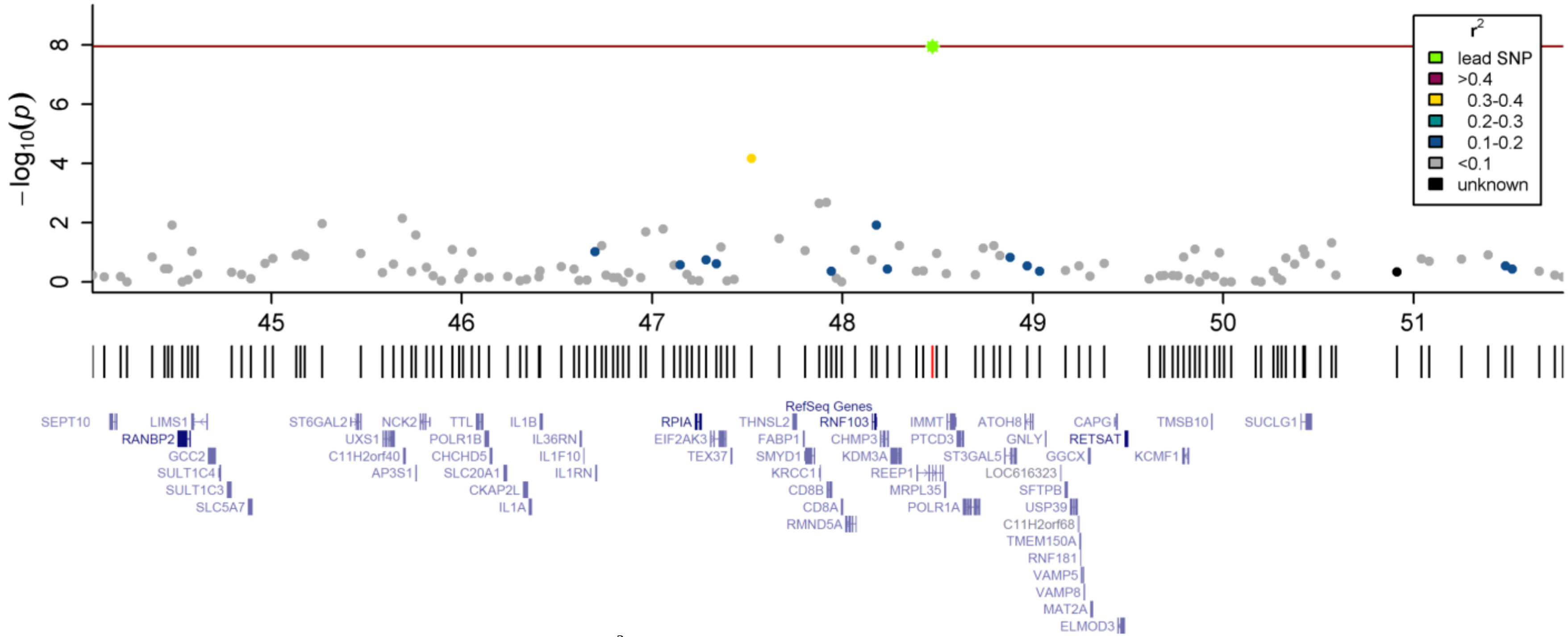

Supplementary Figure S1. Pairwise linkage disequilibrium (r^2) between the leading single nucleotide polymorphism (lead SNP) and its adjacent markers at their mega base pair positions on chromosome 11. They are shown in relation to their $-\log_{10}$ *p*-values, which were calculated by regressing parent-of-origin effects of parents estimated in net body weight gain on their genotypes when the *c*-parameter equaled 0.1. The red line corresponds to a genome-wide false discovery rate of 5%. Genes are displayed depending on their physical positions (Bos Taurus UMD3.1.1/bosTau8; UCSC Genome Browser; Assembly date: Dec. 2009; http://genome.ucsc.edu/ [last accessed in July 2017]). The red bars indicate significant SNPs.

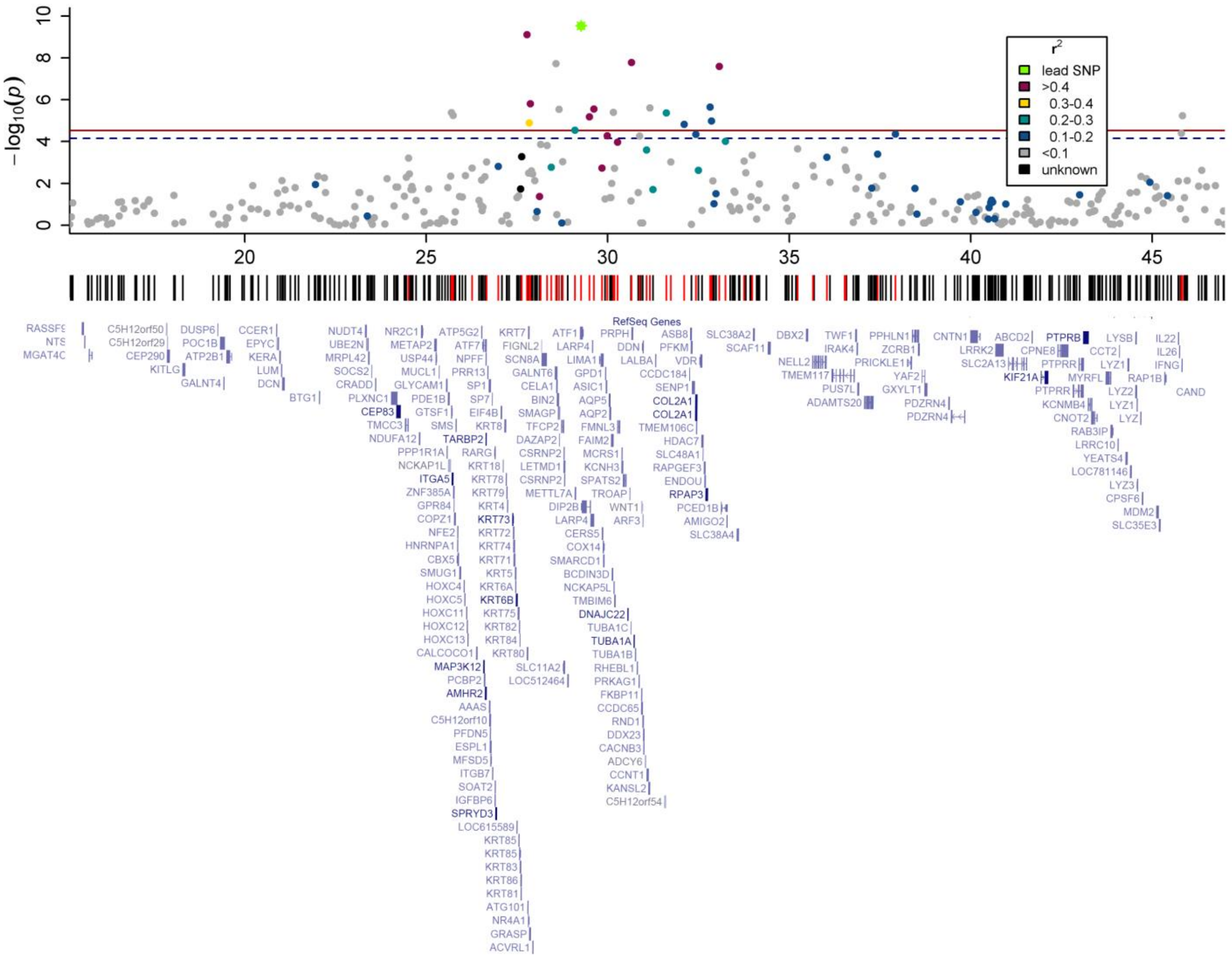

Supplementary Figure S2. Pairwise linkage disequilibrium (r^2) between the leading single nucleotide polymorphism (lead SNP) and its adjacent markers at their mega base pair positions on chromosome 5. They are shown in relation to their $-\log_{10}$ *p*-values, which were calculated by regressing transmitting abilities as sire estimated in carcass fatness (analysed via a linear model) on genotypes. The red line corresponds to a genome-wide false discovery rate of 5%. The blue dashed line corresponds to a genome-wide false discovery rate of 10%. Genes are displayed depending on their physical positions (Bos Taurus UMD3.1.1/bosTau8; UCSC Genome Browser; Assembly date: Dec. 2009; http://genome.ucsc.edu/ [last accessed in July 2017]). The red bars indicate significant SNPs.

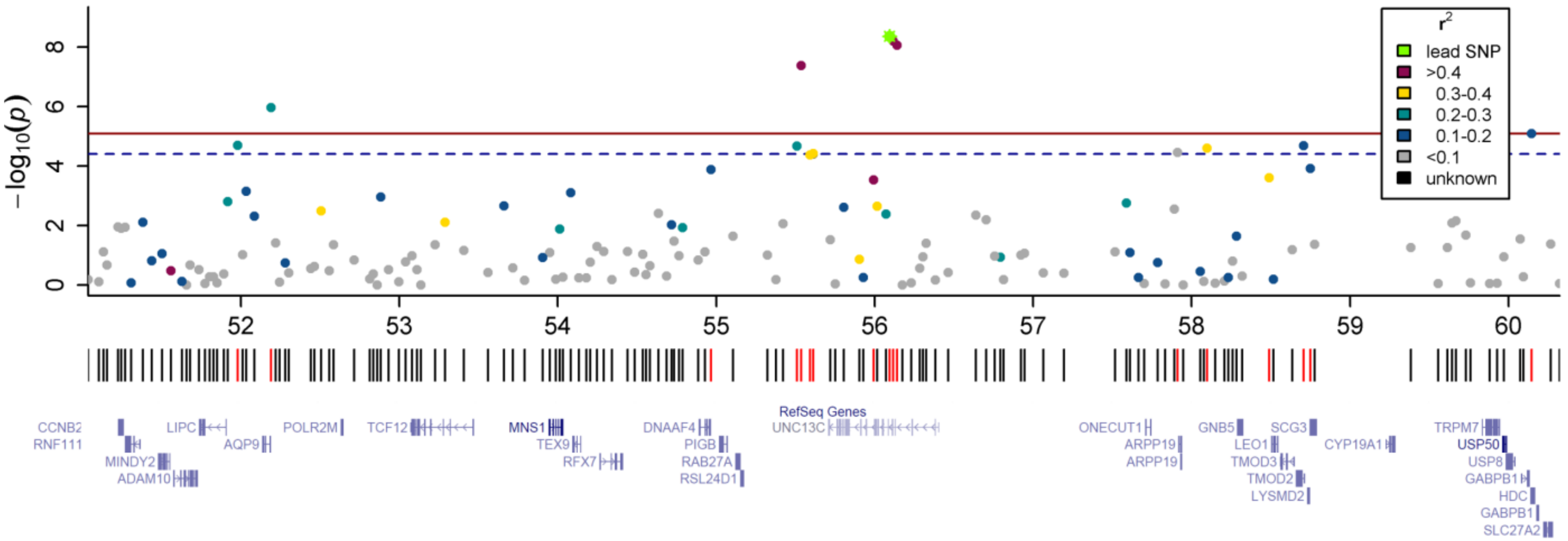

Supplementary Figure S3. Pairwise linkage disequilibrium (r^2) between the leading single nucleotide polymorphism (lead SNP) and its adjacent markers at their mega base pair positions on chromosome 10. They are shown in relation to their $-\log_{10}$ *p*-values, which were calculated by regressing transmitting abilities as sire estimated in muscularity (analysed via a linear model) on genotypes. The red line corresponds to a genome-wide false discovery rate of 5%. The blue dashed line corresponds to a genome-wide false discovery rate of 10%. Genes are displayed depending on their physical positions (Bos Taurus UMD3.1.1/bosTau8; UCSC Genome Browser; Assembly date: Dec. 2009; http://genome.ucsc.edu/ [last accessed in July 2017]). The red bars indicate significant SNPs.

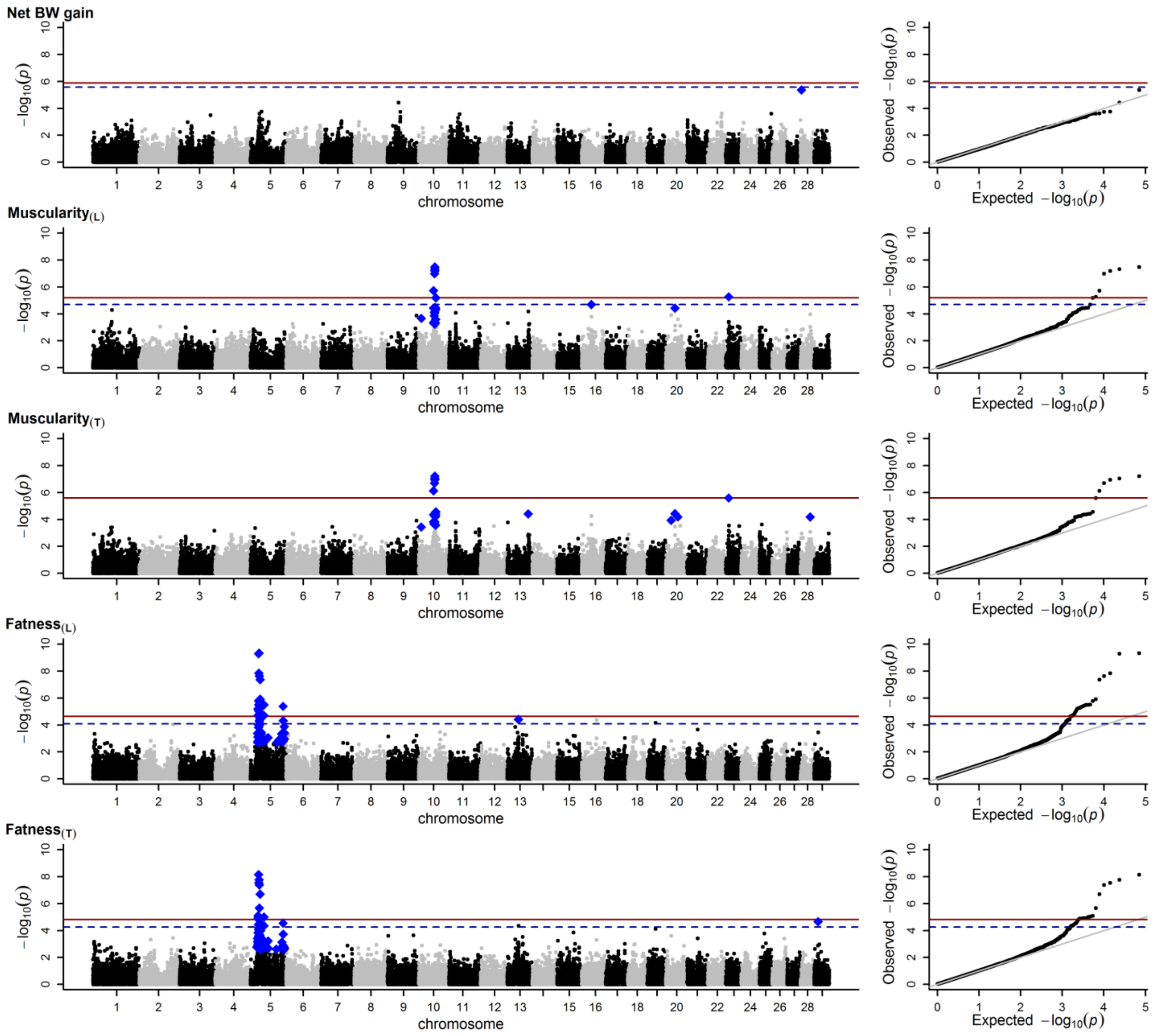

Supplementary Figure S4. Marker loci in relation to their $-\log_{10}$ *p*-values generated by regressing transmitting abilities as dam (TAs) on marker genotypes. The TAs for carcass muscularity and carcass fatness were generated using a linear (subscript L) and threshold model (subscript T). Red line = 5% genome-wide false discovery rate (FDR); dashed line = 10% genome-wide FDR; blue diamonds = markers significant assuming a chromosome-wide FDR of 5%.

Supplementary Tables

Supplementary Table S1. Average marker effects (*mean*) together with their standard deviations between repeated simulations (*sd*) and average standard errors (*se*) for all analyses and markers linked to quantitative trait loci (QTLs) with biparental (Men), paternal (Pat), maternal (Mat), partial paternal (Pat/Men) and partial maternal (Mat/Men) expression patterns. For markers not linked to QTLs (others), ranges are given. Subscripts *imp* and *add* indicate separate tests related to imprinting, or Mendelian effects, while *mkr* indicates an unspecified single effect marker test from model 2B. Estimates of the parent's parent-of-origin effects (1A) and transmitting abilities (1B) were regressed on their un-ordered genotype; phenotypes of offspring were regressed on their own ordered (2A) and un-ordered genotype (2B). Estimated parent-of-origin effects were analysed untreated (3A), deregressed and corrected for parent average (3B), and deregressed, corrected for parent-average and weighted (3C).

Analysis		Marker and expression pattern					
		2	5	8	11	14	others
		Men	Pat	Mat	Pat/Men	Mat/Men	no
1A	$mean_{imp}$	0.004	−0.176	0.169	−0.181	0.178	−0.002 – 0.007
	sd_{imp}	0.053	0.054	0.054	0.049	0.053	0.044 – 0.054
	$\pm se_{imp}$	0.049	0.049	0.049	0.049	0.049	0.049 – 0.055
1B	$mean_{add}$	0.272	0.086	0.082	0.320	0.333	−0.013 – 0.017
	sd_{add}	0.074	0.082	0.078	0.077	0.073	0.067 – 0.099
	$\pm se_{add}$	0.071	0.081	0.080	0.074	0.074	0.076 – 0.085
2A	$mean_{add}$	0.469	0.158	0.149	0.534	0.542	−0.004 – 0.002
	sd_{add}	0.056	0.024	0.025	0.044	0.043	0.018 – 0.022
	$\pm se_{add}$	0.019	0.020	0.020	0.020	0.020	0.019 – 0.021
2A	$mean_{imp}$	0.000	0.167	−0.152	0.169	−0.170	−0.003 – 0.004
	sd_{imp}	0.025	0.023	0.027	0.024	0.029	0.023 – 0.030
	$\pm se_{imp}$	0.024	0.024	0.024	0.024	0.024	0.024 – 0.025
2B	$mean_{mkr}$	0.469	0.158	0.149	0.534	0.543	−0.004 – 0.002
	sd_{mkr}	0.056	0.024	0.025	0.045	0.043	0.018 – 0.022
	$\pm se_{mkr}$	0.019	0.020	0.020	0.020	0.020	0.019 – 0.021
3A	$mean_{imp}$	0.003	−0.082	0.077	−0.082	0.087	−0.003 – 0.003
	sd_{imp}	0.023	0.035	0.037	0.036	0.034	0.016 – 0.023
	$\pm se_{imp}$	0.017	0.018	0.019	0.017	0.018	0.017 – 0.019
3B	$mean_{imp}$	0.005	−0.136	0.126	−0.135	0.143	−0.005 – 0.004
	sd_{imp}	0.038	0.058	0.061	0.061	0.056	0.026 – 0.037
	$\pm se_{imp}$	0.028	0.030	0.031	0.028	0.029	0.030 – 0.032
3C	$mean_{imp}$	0.005	−0.135	0.126	−0.135	0.142	−0.005 – 0.004
	sd_{imp}	0.037	0.058	0.061	0.060	0.056	0.026 – 0.037
	$\pm se_{imp}$	0.027	0.029	0.030	0.028	0.028	0.027 – 0.031

Supplementary Table S2. Single nucleotide polymorphisms (SNPs) significantly associated with transmitting abilities as sire (TAs) estimated for net body weight (BW) gain, carcass muscularity and carcass fatness. The TAs for carcass muscularity and carcass fatness were generated using a linear (subscript L) and threshold model (subscript T). The estimated SNP effects are provided with standard errors (se) and *p*-values. Their base pair (bp) positions on chromosomes (chr) and known genes are indicated.

Trait	SNP	effect	se	*p*-value	chr	bp	gene
Net BW gain	Hapmap49247-BTA-63647	−8.3648	1.8187	4.2470e-06*	28	3637555	
Muscularity$_{(L)}$	BTB-00429961	−1.2325	0.2099	4.3065e-09***	10	56093653	UNC13C
	ARS-BFGL-NGS-21100	−1.2186	0.2098	6.2669e-09***	10	56116909	UNC13C
	BTB-01911175	1.2083	0.2100	8.7539e-09***	10	56140822	UNC13C
	BTB-00430730	1.1607	0.2118	4.2323e-08***	10	55535781	
	Hapmap51030-BTA-69263	1.2283	0.2521	1.0970e-06***	10	52190618	AQP9
	Hapmap50767-BTA-72346	1.2731	0.2852	8.0751e-06***	10	60145660	HDC
	ARS-BFGL-NGS-24556	−0.8659	0.2029	1.9888e-05**	10	51980270	
	Hapmap36252-SCAFFOLD195517_10504	−1.0007	0.2352	2.0960e-05**	10	58707718	TMOD2
	ARS-BFGL-NGS-27708	−0.8953	0.2105	2.1071e-05**	10	55510249	
	Hapmap58597-rs29013533	−0.8634	0.2049	2.5192e-05**	10	58097440	
	BTB-01125630	1.5872	0.3834	3.4723e-05**	10	57912228	
	Hapmap59786-rs29012019	−0.8747	0.2123	3.7975e-05**	10	55611885	
	ARS-USMARC-Parent-DQ984827-rs29012019	−0.8721	0.2121	3.9402e-05**	10	55611885	
	BTB-00428180	0.8700	0.2126	4.2418e-05*	10	55591993	
	BTA-114684-no-rs	0.8611	0.2241	1.2210e-04*	10	58749431	SCG3
	BTB-00429005	1.1514	0.3010	1.3082e-04*	10	54968099	DNAAF4
	ARS-BFGL-NGS-55539	0.8171	0.2231	2.5029e-04*	10	58488593	
	BTB-00430147	−0.7769	0.2144	2.9061e-04*	10	55993255	UNC13C
	ARS-BFGL-NGS-55845	0.7200	0.2013	3.4662e-04*	10	9821501	ARSB
	ARS-BFGL-NGS-31807	−0.7336	0.2106	4.9617e-04*	10	61684704	
	ARS-BFGL-NGS-114479	1.1821	0.2798	2.3777e-05**	16	33402551	
	ARS-BFGL-NGS-40515	−0.9244	0.2048	6.3823e-06***	23	10593430	
Muscularity$_{(T)}$	BTB-00429961	0.1888	0.0317	2.6987e-09***	10	56093653	UNC13C
	ARS-BFGL-NGS-21100	0.1865	0.0317	4.0909e-09***	10	56116909	UNC13C
	BTB-01911175	−0.1851	0.0318	5.5682e-09***	10	56140822	UNC13C
	BTB-00430730	−0.1770	0.0320	3.0586e-08***	10	55535781	
	Hapmap51030-BTA-69263	−0.1963	0.0383	2.9533e-07***	10	52190618	AQP9
	Hapmap36252-SCAFFOLD195517_10504	0.1532	0.0355	1.5870e-05**	10	58707718	TMOD2

Trait	SNP	effect	se	*p*-value	chr	bp	gene
	ARS-BFGL-NGS-24556	0.1315	0.0307	1.8382e-05**	10	51980270	
	ARS-BFGL-NGS-27708	0.1359	0.0318	1.9373e-05**	10	55510249	
	Hapmap50767-BTA-72346	−0.1794	0.0433	3.4541e-05*	10	60145660	HDC
	BTB-00429005	−0.1866	0.0457	4.4479e-05*	10	54968099	DNAAF4
	BTB-01125630	−0.2336	0.0580	5.6099e-05*	10	57912228	
	Hapmap58597-rs29013533	0.1246	0.0310	5.7296e-05*	10	58097440	
	BTB-00428180	−0.1284	0.0322	6.5038e-05*	10	55591993	
	Hapmap59786-rs29012019	0.1283	0.0321	6.5038e-05*	10	55611885	
	ARS-USMARC-Parent-DQ984827-rs29012019	0.1275	0.0321	7.0776e-05*	10	55611885	
	BTA-114684-no-rs	−0.1314	0.0338	1.0305e-04*	10	58749431	SCG3
	BTB-00430147	0.1178	0.0324	2.7698e-04*	10	55993255	UNC13C
	ARS-BFGL-NGS-5130	0.1788	0.0494	2.9061e-04*	10	52033595	
	ARS-BFGL-NGS-55845	−0.1055	0.0304	5.2633e-04*	10	9821501	ARSB
	BTA-95978-no-rs	0.1696	0.0493	5.7661e-04*	10	54082665	
	ARS-BFGL-NGS-55539	−0.1154	0.0337	6.2165e-04*	10	58488593	
	ARS-BFGL-NGS-31807	0.1083	0.0318	6.6307e-04*	10	61684704	
	ARS-BFGL-NGS-40515	0.1409	0.0308	4.6169e-06***	23	10593430	
	ARS-BFGL-NGS-11408	0.1514	0.0400	1.5670e-04*	23	11007995	
	Hapmap49110-BTA-29232	0.1478	0.0394	1.7151e-04*	23	12519690	
	ARS-BFGL-NGS-88771	−0.1243	0.0310	6.1044e-05*	28	33359220	KCNMA1
Fatness$_{(L)}$	ARS-BFGL-NGS-67309	0.0240	0.0057	2.5727e-05***	2	114710664	
	ARS-BFGL-NGS-38020	0.0328	0.0052	2.9461e-10***	5	29264678	
	Hapmap39353-BTA-73120	−0.0335	0.0055	7.9597e-10***	5	27777281	
	ARS-BFGL-NGS-53461	0.0292	0.0052	1.7002e-08***	5	30659497	TROAP
	ARS-BFGL-NGS-91751	−0.0332	0.0059	1.9337e-08***	5	28573792	
	ARS-BFGL-NGS-92420	0.0293	0.0053	2.5670e-08***	5	33078266	
	ARS-BFGL-NGS-30168	0.0266	0.0054	8.8196e-07***	5	111313740	SYNGR1
	ARS-BFGL-NGS-98156	0.0255	0.0053	1.5540e-06***	5	27869236	
	Hapmap42150-BTA-25138	−0.0257	0.0054	2.2840e-06***	5	32819286	
	ARS-USMARC-675	0.0410	0.0087	2.5085e-06***	5	31164990	ADCY6
	Hapmap51043-BTA-73218	0.0230	0.0049	2.8574e-06***	5	29622395	LARP4
	ARS-BFGL-NGS-107085	−0.0330	0.0071	2.9176e-06***	5	28660813	BIN2

Trait	SNP	effect	se	*p*-value	chr	bp	gene
	ARS-BFGL-NGS-112542	0.0288	0.0063	4.0947e-06***	5	30159843	FAIM2
	BTA-72978-no-rs	−0.0274	0.0060	4.2470e-06***	5	25699752	NCKAP1L
	Hapmap23022-BTA-161235	−0.0245	0.0053	4.2916e-06***	5	31613026	
	ARS-USMARC-629	0.0310	0.0068	5.6301e-06***	5	25738874	GTSF1
	ARS-USMARC-657	0.0281	0.0062	5.8398e-06***	5	45834943	IFNG
	BTA-73209-no-rs	0.0230	0.0051	6.6548e-06***	5	29496625	DIP2B
	ARS-BFGL-NGS-68582	−0.0251	0.0056	7.7038e-06***	5	111380409	
	ARS-BFGL-NGS-717	0.0240	0.0055	1.0656e-05***	5	32861996	
	ARS-BFGL-NGS-7725	−0.0220	0.0051	1.3210e-05***	5	27839649	KRT7
	BTA-73392-no-rs	0.0243	0.0056	1.4981e-05***	5	32104484	
	ARS-BFGL-NGS-8796	0.0227	0.0054	2.9035e-05***	5	29095603	
	ARS-USMARC-652	0.0282	0.0069	4.0453e-05**	5	45807839	
	BTA-28787-no-rs	0.0221	0.0054	4.4479e-05**	5	37925443	
	ARS-BFGL-NGS-11851	0.0226	0.0055	4.5188e-05**	5	32426908	
	UA-IFASA-2781	0.0218	0.0054	5.3496e-05**	5	29989860	ASIC1
	ARS-BFGL-NGS-43909	−0.0282	0.0070	5.4350e-05**	5	30879776	
	Hapmap46916-BTA-105154	−0.0220	0.0056	9.7217e-05*	5	33246646	PCED1B
	Hapmap39286-BTA-73191	−0.0210	0.0054	1.0866e-04*	5	30275164	
	ARS-BFGL-NGS-100971	0.0287	0.0075	1.3942e-04*	5	28153566	
	ARS-BFGL-NGS-7567	−0.0242	0.0064	1.5504e-04*	5	28331294	SCN8A
	ARS-BFGL-NGS-93481	0.0241	0.0064	1.6525e-04*	5	115223183	
	Hapmap56154-rs29018650	0.0379	0.0103	2.2861e-04*	5	35234997	
	ARS-BFGL-NGS-104808	−0.0223	0.0061	2.3478e-04*	5	111635921	
	ARS-BFGL-NGS-29931	−0.0189	0.0052	2.5162e-04*	5	36528436	TMEM117
	BTA-111859-no-rs	0.0267	0.0073	2.5297e-04*	5	91044792	
	ARS-BFGL-NGS-112163	−0.0191	0.0052	2.5568e-04*	5	31074760	
	BTB-01248388	0.0198	0.0056	4.0262e-04*	5	37440296	
	BTB-01495858	−0.0204	0.0058	4.5784e-04*	5	33981125	
	BTA-05125-rs29019289	−0.0191	0.0055	4.8564e-04*	5	90866134	
	ARS-BFGL-NGS-113311	−0.0195	0.0056	5.3201e-04*	5	27633265	KRT75
	ARS-BFGL-NGS-40375	−0.0187	0.0054	5.5833e-04*	5	36041533	NELL2
	Hapmap58633-rs29009713	0.0202	0.0059	6.0843e-04*	5	97828652	BORCS5

Trait	SNP	effect	se	*p*-value	chr	bp	gene
	ARS-BFGL-NGS-10291	0.0203	0.0059	6.1832e-04*	5	24512405	TMCC3
	ARS-BFGL-NGS-111020	−0.0172	0.0051	6.9596e-04*	5	106558376	
	ARS-BFGL-NGS-33993	0.0176	0.0052	7.6677e-04*	5	107031270	TSPAN11
	BTB-01205531	−0.0204	0.0061	8.6795e-04*	5	33762720	
	ARS-BFGL-NGS-115973	−0.0323	0.0098	9.2599e-04*	5	28757093	
	ARS-BFGL-NGS-118817	−0.0263	0.0080	9.4110e-04*	5	31739928	
	ARS-BFGL-NGS-34382	0.0340	0.0104	9.5132e-04*	5	26661043	ATF7
	Hapmap33270-BTA-75139	0.0300	0.0091	1.0205e-03*	5	112559317	
	ARS-BFGL-NGS-100028	−0.0183	0.0057	1.1682e-03*	5	86753605	SOX5
	BTA-21377-no-rs	−0.0237	0.0074	1.4120e-03*	5	35657214	
	Hapmap41340-BTA-71012	−0.0213	0.0067	1.4197e-03*	5	115693793	
	ARS-BFGL-NGS-5790	−0.0174	0.0055	1.5318e-03*	5	26986116	
	ARS-BFGL-NGS-89539	0.0178	0.0056	1.5739e-03*	5	50175236	
	ARS-BFGL-NGS-72724	−0.0152	0.0048	1.5911e-03*	5	116005043	
	Hapmap41951-BTA-73168	0.0176	0.0056	1.6891e-03*	5	28442563	
	BTB-02015191	0.0166	0.0053	1.7169e-03*	5	36553084	TMEM117
	ARS-BFGL-NGS-21191	−0.0297	0.0096	1.8730e-03*	5	26263552	
	ARS-BFGL-NGS-20135	0.0170	0.0055	1.8832e-03*	5	29839619	LIMA1
	ARS-BFGL-NGS-119788	−0.0221	0.0071	1.9458e-03*	5	30185840	
	ARS-BFGL-NGS-55084	−0.0171	0.0055	1.9778e-03*	5	60513092	
	Hapmap55179-rs29024483	0.0231	0.0055	2.9496e-05***	13	38830375	DZANK1
	Hapmap55095-rs29010810	0.0260	0.0065	7.0403e-05**	13	27589301	
	ARS-BFGL-NGS-4866	−0.0317	0.0079	5.3215e-05**	16	50095343	
	ARS-BFGL-NGS-18515	0.0231	0.0055	2.2798e-05***	19	29219211	NTN1
$\text{Fatness}_{(T)}$	Hapmap39353-BTA-73120	−0.1948	0.0342	1.1676e-08***	5	27777281	
	ARS-BFGL-NGS-38020	0.1858	0.0326	1.1858e-08***	5	29264678	
	ARS-BFGL-NGS-91751	−0.2030	0.0368	3.5517e-08***	5	28573792	
	ARS-BFGL-NGS-53461	0.1769	0.0324	4.7653e-08***	5	30659497	TROAP
	ARS-BFGL-NGS-92420	0.1746	0.0329	1.0884e-07***	5	33078266	
	ARS-USMARC-629	0.1985	0.0427	3.3064e-06***	5	25738874	GTSF1
	ARS-BFGL-NGS-112542	0.1814	0.0391	3.5568e-06***	5	30159843	FAIM2
	ARS-BFGL-NGS-30168	0.1530	0.0339	6.5171e-06***	5	111313740	SYNGR1

Trait	SNP	effect	se	*p*-value	chr	bp	gene
	ARS-BFGL-NGS-107085	−0.1945	0.0442	1.0769e-05***	5	28660813	BIN2
	ARS-USMARC-675	0.2390	0.0544	1.0997e-05***	5	31164990	ADCY6
	BTA-72978-no-rs	−0.1631	0.0372	1.1527e-05***	5	25699752	NCKAP1L
	ARS-BFGL-NGS-43909	−0.1900	0.0436	1.3004e-05***	5	30879776	
	Hapmap42150-BTA-25138	−0.1466	0.0338	1.4670e-05***	5	32819286	
	ARS-BFGL-NGS-8796	0.1442	0.0338	2.0420e-05**	5	29095603	
	ARS-USMARC-657	0.1661	0.0390	2.0850e-05**	5	45834943	IFNG
	ARS-BFGL-NGS-68582	−0.1481	0.0351	2.3777e-05**	5	111380409	
	ARS-BFGL-NGS-7725	−0.1324	0.0317	2.9808e-05**	5	27839649	KRT7
	ARS-BFGL-NGS-98156	0.1379	0.0332	3.2770e-05**	5	27869236	
	Hapmap51043-BTA-73218	0.1282	0.0309	3.4000e-05**	5	29622395	LARP4
	BTA-73209-no-rs	0.1310	0.0320	4.1973e-05**	5	29496625	DIP2B
	ARS-BFGL-NGS-717	0.1386	0.0338	4.1973e-05**	5	32861996	
	Hapmap56154-rs29018650	0.2576	0.0638	5.3779e-05**	5	35234997	
	Hapmap23022-BTA-161235	−0.1308	0.0334	9.1717e-05*	5	31613026	
	ARS-USMARC-652	0.1676	0.0431	9.9826e-05*	5	45807839	
	ARS-BFGL-NGS-100971	0.1828	0.0472	1.0582e-04*	5	28153566	
	ARS-BFGL-NGS-7567	−0.1496	0.0400	1.8477e-04*	5	28331294	SCN8A
	ARS-BFGL-NGS-29931	−0.1184	0.0324	2.5568e-04*	5	36528436	TMEM117
	UA-IFASA-2781	0.1226	0.0337	2.7550e-04*	5	29989860	ASIC1
	Hapmap33270-BTA-75139	0.2046	0.0567	3.0983e-04*	5	112559317	
	ARS-BFGL-NGS-11851	0.1252	0.0348	3.1652e-04*	5	32426908	
	ARS-BFGL-NGS-115973	−0.2171	0.0611	3.8369e-04*	5	28757093	
	BTB-01205531	−0.1353	0.0382	3.9199e-04*	5	33762720	
	ARS-BFGL-NGS-112163	−0.1148	0.0328	4.6030e-04*	5	31074760	
	BTA-73392-no-rs	0.1228	0.0351	4.6277e-04*	5	32104484	
	ARS-BFGL-NGS-33993	0.1143	0.0328	4.8825e-04*	5	107031270	TSPAN11
	BTA-28787-no-rs	0.1165	0.0338	5.7045e-04*	5	37925443	
	ARS-BFGL-NGS-40375	−0.1146	0.0337	6.8115e-04*	5	36041533	NELL2
	BTB-01495858	−0.1227	0.0363	7.2658e-04*	5	33981125	
	BTB-01205481	−0.1301	0.0386	7.4640e-04*	5	33808700	
	Hapmap46916-BTA-105154	−0.1187	0.0353	7.5855e-04*	5	33246646	PCED1B

Trait	SNP	effect	se	*p*-value	chr	bp	gene
	ARS-BFGL-NGS-10291	0.1244	0.0372	8.1797e-04*	5	24512405	TMCC3
	ARS-BFGL-NGS-89539	0.1176	0.0352	8.3131e-04*	5	50175236	
	ARS-BFGL-NGS-119788	−0.1490	0.0446	8.4486e-04*	5	30185840	
	BTA-54940-no-rs	−0.1084	0.0325	8.4486e-04*	5	55263796	
	ARS-BFGL-NGS-104808	−0.1265	0.0380	8.5864e-04*	5	111635921	
	Hapmap41346-BTA-72920	0.1175	0.0356	9.4620e-04*	5	22196364	
	ARS-USMARC-Parent-DQ500958-no-rs	−0.1225	0.0371	9.7208e-04*	5	27825118	
	ARS-BFGL-NGS-113311	−0.1150	0.0350	1.0041e-03*	5	27633265	KRT75
	ARS-BFGL-NGS-5790	−0.1123	0.0342	1.0316e-03*	5	26986116	
	Hapmap41950-BTA-72999	−0.1158	0.0354	1.0541e-03*	5	26082666	
	Hapmap39286-BTA-73191	−0.1111	0.0339	1.0541e-03*	5	30275164	
	ARS-BFGL-NGS-66566	−0.1279	0.0393	1.1248e-03*	5	36122701	
	BTA-75143-no-rs	−0.1143	0.0357	1.3595e-03*	5	112647134	XPNPEP3
	ARS-BFGL-NGS-52457	−0.1167	0.0365	1.3818e-03*	5	104116518	PIANP
	ARS-BFGL-NGS-34382	0.2074	0.0651	1.4509e-03*	5	26661043	ATF7
	ARS-BFGL-NGS-34352	0.1064	0.0336	1.5318e-03*	5	49842685	SRGAP1
	ARS-BFGL-NGS-93481	0.1251	0.0401	1.8227e-03*	5	115223183	
	Hapmap43887-BTA-58386	−0.1004	0.0323	1.8527e-03*	5	54045462	SLC16A7
	ARS-BFGL-NGS-21191	−0.1858	0.060	1.8935e-03*	5	26263552	
	ARS-BFGL-NGS-77906	−0.1070	0.0345	1.9352e-03*	5	24533402	TMCC3
	ARS-BFGL-NGS-55084	−0.1069	0.0345	1.9352e-03*	5	60513092	
	Hapmap47087-BTA-73116	−0.1809	0.0584	1.9564e-03*	5	27898166	KRT80
	ARS-BFGL-NGS-111020	−0.0980	0.0318	2.0436e-03*	5	106558376	
	BTA-72912-no-rs	−0.1519	0.0494	2.0886e-03*	5	22020313	
	ARS-BFGL-NGS-110506	−0.1053	0.0342	2.0886e-03*	5	105152085	NTF3
	Hapmap55179-rs29024483	0.1452	0.0344	2.4928e-05**	13	38830375	DZANK1
	ARS-BFGL-NGS-18515	0.1445	0.0342	2.4410e-05**	19	29219211	NTN1

***5% genome-wide false discovery rate; **10% genome-wide false discovery rate; *5% chromosome-wide false discovery rate

Supplementary Table S3. Single nucleotide polymorphisms (SNPs) significantly associated with transmitting abilities as dam (TAs) estimated for net body weight (BW) gain, carcass muscularity and carcass fatness. The TAs for carcass muscularity and carcass fatness were generated using a linear (subscript L) and threshold model (subscript T). The estimated SNP effects are provided with standard errors (se) and *p*-values. Their base pair (bp) positions on chromosomes (chr) and known genes are indicated.

Trait	SNP	effect	se	*p*-value	chr	bp	gene
Net BW gain	Hapmap49247-BTA-63647	−9.7952	2.1293	4.2249e-06*	28	3637555	
Muscularity$_{(L)}$	BTB-00429961	−1.3037	0.2360	3.3044e-08***	10	56093653	UNC13C
	ARS-BFGL-NGS-21100	−1.2881	0.2358	4.7164e-08***	10	56116909	UNC13C
	BTB-01911175	1.2778	0.2361	6.2317e-08***	10	56140822	UNC13C
	BTB-00430730	1.2633	0.2375	1.0443e-07***	10	55535781	
	Hapmap51030-BTA-69263	1.3499	0.2830	1.8450e-06***	10	52190618	AQP9
	Hapmap50767-BTA-72346	1.4423	0.3197	6.4493e-06***	10	60145660	HDC
	Hapmap58597-rs29013533	−0.9521	0.2295	3.3467e-05*	10	58097440	
	ARS-BFGL-NGS-27708	−0.9781	0.2363	3.4723e-05*	10	55510249	
	ARS-BFGL-NGS-24556	−0.9369	0.2272	3.7380e-05*	10	51980270	
	BTB-01125630	1.7619	0.4291	4.0241e-05*	10	57912228	
	Hapmap36252-SCAFFOLD195517_10504	−1.0755	0.2645	4.7886e-05*	10	58707718	TMOD2
	Hapmap59786-rs29012019	−0.9452	0.2383	7.2671e-05*	10	55611885	
	ARS-USMARC-Parent-DQ984827-rs29012019	−0.9413	0.2381	7.7023e-05*	10	55611885	
	BTB-00428180	0.9387	0.2390	8.3383e-05*	10	55591993	
	BTB-00429005	1.2890	0.3379	1.3649e-04*	10	54968099	DNAAF4
	ARS-BFGL-NGS-55845	0.8355	0.2254	2.0994e-04*	10	9821501	ARSB
	BTA-114684-no-rs	0.9208	0.2515	2.5162e-04*	10	58749431	SCG3
	BTA-95978-no-rs	−1.2853	0.3600	3.9833e-04*	10	54082665	
	ARS-BFGL-NGS-55539	0.8760	0.2498	4.5296e-04*	10	58488593	
	ARS-BFGL-NGS-5130	−1.2770	0.3649	4.6526e-04*	10	52033595	
	BTB-00430147	−0.8299	0.2407	5.6436e-04*	10	55993255	UNC13C
	ARS-BFGL-NGS-114479	1.3420	0.3145	1.9784e-05**	16	33402551	
	ARS-BFGL-NGS-10108	0.9655	0.2341	3.7184e-05*	20	31848979	
	ARS-BFGL-NGS-40515	−1.0428	0.2293	5.4280e-06***	23	10593430	
Muscularity$_{(T)}$	BTB-00429961	0.2219	0.0410	6.0106e-08***	10	56093653	UNC13C
	ARS-BFGL-NGS-21100	0.2189	0.0409	8.8069e-08***	10	56116909	UNC13C
	BTB-01911175	−0.2174	0.0410	1.1111e-07***	10	56140822	UNC13C
	BTB-00430730	−0.2141	0.0412	2.0136e-07***	10	55535781	

Trait	SNP	effect	se	p-value	chr	bp	gene
	Hapmap51030-BTA-69263	−0.2447	0.0494	7.4310e-07***	10	52190618	AQP9
	Hapmap50767-BTA-72346	−0.2340	0.0557	2.6974e-05*	10	60145660	HDC
	Hapmap36252-SCAFFOLD195517_10504	0.1881	0.0460	4.2868e-05*	10	58707718	TMOD2
	ARS-BFGL-NGS-24556	0.1613	0.0394	4.3094e-05*	10	51980270	
	BTB-00429005	−0.2396	0.0588	4.6640e-05*	10	54968099	DNAAF4
	ARS-BFGL-NGS-27708	0.1656	0.0410	5.4350e-05*	10	55510249	
	BTB-01125630	−0.2993	0.0744	5.7296e-05*	10	57912228	
	Hapmap58597-rs29013533	0.1594	0.0398	6.1367e-05*	10	58097440	
	Hapmap59786-rs29012019	0.1571	0.0414	1.4547e-04*	10	55611885	
	BTB-00428180	−0.1573	0.0414	1.4624e-04*	10	55591993	
	ARS-BFGL-NGS-5130	0.2405	0.0634	1.4859e-04*	10	52033595	
	ARS-USMARC-Parent-DQ984827-rs29012019	0.1559	0.0413	1.6263e-04*	10	55611885	
	BTA-95978-no-rs	0.2333	0.0631	2.1675e-04*	10	54082665	
	BTA-114684-no-rs	−0.1591	0.0440	2.6259e-04*	10	58749431	SCG3
	ARS-BFGL-NGS-55845	−0.1399	0.0392	3.6177e-04*	10	9821501	ARSB
	Hapmap54667-rs29016013	0.2172	0.0527	3.7975e-05*	13	72431970	
	ARS-BFGL-NGS-10108	−0.1674	0.0405	3.6408e-05*	20	31848979	
	ARS-BFGL-BAC-33671	−0.1595	0.0399	6.4015e-05*	20	41576197	
	ARS-BFGL-NGS-25782	0.2377	0.0617	1.1518e-04*	20	18537789	
	ARS-BFGL-NGS-40515	0.1866	0.0396	2.4954e-06***	23	10593430	
	ARS-BFGL-NGS-88771	−0.1597	0.0400	6.5729e-05*	28	33359220	KCNMA1
Fatness$_{(L)}$	ARS-BFGL-NGS-38020	0.0362	0.0058	4.5299e-10***	5	29264678	
	Hapmap39353-BTA-73120	−0.0378	0.0061	4.9168e-10***	5	27777281	
	ARS-BFGL-NGS-91751	−0.0373	0.0066	1.3981e-08***	5	28573792	
	ARS-BFGL-NGS-53461	0.0323	0.0058	2.2920e-08***	5	30659497	TROAP
	ARS-BFGL-NGS-92420	0.0321	0.0059	4.2982e-08***	5	33078266	
	Hapmap42150-BTA-25138	−0.0294	0.0061	1.2171e-06***	5	32819286	
	ARS-BFGL-NGS-98156	0.0283	0.0059	1.6542e-06***	5	27869236	
	ARS-BFGL-NGS-107085	−0.0367	0.0079	3.0737e-06***	5	28660813	BIN2
	ARS-USMARC-657	0.0323	0.0069	3.1059e-06***	5	45834943	IFNG
	Hapmap23022-BTA-161235	−0.0278	0.0060	3.1879e-06***	5	31613026	
	ARS-BFGL-NGS-112542	0.0323	0.0070	3.6508e-06***	5	30159843	FAIM2

Trait	SNP	effect	se	*p*-value	chr	bp	gene
	ARS-BFGL-NGS-30168	0.0279	0.0061	4.1161e-06***	5	111313740	SYNGR1
	ARS-USMARC-675	0.0445	0.0097	4.5928e-06***	5	31164990	ADCY6
	Hapmap51043-BTA-73218	0.0250	0.0055	5.3997e-06***	5	29622395	LARP4
	ARS-BFGL-NGS-717	0.0276	0.0061	5.4850e-06***	5	32861996	
	BTA-72978-no-rs	−0.0300	0.0066	6.3823e-06***	5	25699752	NCKAP1L
	ARS-BFGL-NGS-7725	−0.0251	0.0056	8.6887e-06***	5	27839649	KRT7
	BTA-73392-no-rs	0.0275	0.0063	1.1527e-05***	5	32104484	
	BTA-73209-no-rs	0.0246	0.0057	1.6122e-05***	5	29496625	DIP2B
	ARS-USMARC-652	0.0328	0.0077	1.9577e-05***	5	45807839	
	ARS-USMARC-629	0.0325	0.0076	1.9784e-05***	5	25738874	GTSF1
	ARS-BFGL-NGS-8796	0.0256	0.0060	2.1745e-05***	5	29095603	
	BTA-28787-no-rs	0.0254	0.0060	2.3282e-05***	5	37925443	
	ARS-BFGL-NGS-11851	0.0256	0.0060	3.6601e-05**	5	32426908	
	ARS-BFGL-NGS-43909	−0.0316	0.0078	4.5667e-05**	5	30879776	
	ARS-BFGL-NGS-68582	−0.0255	0.0063	4.8394e-05**	5	111380409	
	UA-IFASA-2781	0.0243	0.0060	5.4637e-05**	5	29989860	ASIC1
	Hapmap46916-BTA-105154	−0.0248	0.0063	8.1206e-05**	5	33246646	PCED1B
	ARS-BFGL-NGS-100971	0.0327	0.0084	9.5685e-05*	5	28153566	
	ARS-BFGL-NGS-7567	−0.0276	0.0071	1.0251e-04*	5	28331294	SCN8A
	Hapmap39286-BTA-73191	−0.0234	0.0061	1.0808e-04*	5	30275164	
	ARS-BFGL-NGS-93481	0.0274	0.0072	1.3151e-04*	5	115223183	
	ARS-BFGL-NGS-113311	−0.0237	0.0063	1.4938e-04*	5	27633265	KRT75
	ARS-BFGL-NGS-104808	−0.0243	0.0068	3.4293e-04*	5	111635921	
	ARS-BFGL-NGS-29931	−0.0206	0.0058	3.5411e-04*	5	36528436	TMEM117
	ARS-BFGL-NGS-72724	−0.0191	0.0054	4.0262e-04*	5	116005043	
	ARS-BFGL-NGS-10291	0.0233	0.0066	4.1799e-04*	5	24512405	TMCC3
	ARS-BFGL-NGS-112163	−0.0206	0.0059	4.2933e-04*	5	31074760	
	ARS-BFGL-NGS-118817	−0.0312	0.0089	4.3629e-04*	5	31739928	
	ARS-BFGL-NGS-111020	−0.0199	0.0057	4.5539e-04*	5	106558376	
	BTB-01248388	0.0217	0.0062	4.7790e-04*	5	37440296	
	BTB-01495858	−0.0225	0.0065	5.2633e-04*	5	33981125	
	ARS-BFGL-NGS-34382	0.0394	0.0116	6.9972e-04*	5	26661043	ATF7

Trait	SNP	effect	se	*p*-value	chr	bp	gene
	Hapmap56154-rs29018650	0.0388	0.0114	7.0349e-04*	5	35234997	
	ARS-BFGL-NGS-5790	−0.0207	0.0061	7.1880e-04*	5	26986116	
	Hapmap41950-BTA-72999	−0.0211	0.0063	8.0486e-04*	5	26082666	
	ARS-BFGL-NGS-55084	−0.0205	0.0062	8.7736e-04*	5	60513092	
	BTB-02015191	0.0197	0.0059	9.0134e-04*	5	36553084	TMEM117
	BTB-01205531	−0.0225	0.0068	9.4110e-04*	5	33762720	
	Hapmap41340-BTA-71012	−0.0244	0.0075	1.0947e-03*	5	115693793	
	BTB-00225371	−0.0192	0.0059	1.1873e-03*	5	36578127	TMEM117
	Hapmap58633-rs29009713	0.0213	0.0066	1.2602e-03*	5	97828652	BORCS5
	ARS-BFGL-NGS-115973	−0.0349	0.0109	1.3303e-03*	5	28757093	
	Hapmap41951-BTA-73168	0.0200	0.0063	1.3521e-03*	5	28442563	
	Hapmap44526-BTA-17619	−0.0197	0.0062	1.4827e-03*	5	50731517	
	ARS-BFGL-NGS-21191	−0.0337	0.0107	1.5485e-03*	5	26263552	
	ARS-BFGL-NGS-15778	−0.0338	0.0107	1.6349e-03*	5	111227019	
	ARS-BFGL-NGS-4809	0.0309	0.0098	1.6349e-03*	5	111682678	ENTHD1
	Hapmap24085-BTA-143102	−0.0404	0.0129	1.7357e-03*	5	46364432	
	ARS-BFGL-NGS-33993	0.0184	0.0059	1.7357e-03*	5	107031270	TSPAN11
	ARS-BFGL-NGS-40375	−0.0189	0.0061	1.8129e-03*	5	36041533	NELL2
	ARS-BFGL-NGS-77906	−0.0192	0.0062	1.8935e-03*	5	24533402	TMCC3
	Hapmap47087-BTA-73116	−0.0324	0.0105	1.9247e-03*	5	27898166	KRT80
	BTA-111859-no-rs	0.0252	0.0081	1.9352e-03*	5	91044792	
	Hapmap55179-rs29024483	0.0253	0.0062	3.9195e-05**	13	38830375	DZANK1
	ARS-BFGL-NGS-4866	−0.0359	0.0088	4.2868e-05**	16	50095343	
	ARS-BFGL-NGS-18515	0.0243	0.0061	6.7846e-05**	19	29219211	NTN1
$Fatness_{(T)}$	Hapmap39353-BTA-73120	−0.2447	0.0423	6.9099e-09***	5	27777281	
	ARS-BFGL-NGS-38020	0.2280	0.0404	1.7002e-08***	5	29264678	
	ARS-BFGL-NGS-91751	−0.2528	0.0456	2.9049e-08***	5	28573792	
	ARS-BFGL-NGS-53461	0.2209	0.0403	4.0822e-08***	5	30659497	TROAP
	ARS-BFGL-NGS-92420	0.2122	0.0408	1.9826e-07***	5	33078266	
	ARS-BFGL-NGS-112542	0.2300	0.0485	2.1124e-06***	5	30159843	FAIM2
	ARS-USMARC-629	0.2372	0.0531	7.8667e-06***	5	25738874	GTSF1
	ARS-BFGL-NGS-107085	−0.2434	0.0548	9.1077e-06***	5	28660813	BIN2

Trait	SNP	effect	se	*p*-value	chr	bp	gene
	ARS-USMARC-657	0.2146	0.0486	1.0007e-05***	5	45834943	IFNG
	Hapmap42150-BTA-25138	−0.1837	0.0420	1.1895e-05***	5	32819286	
	BTA-72978-no-rs	−0.2020	0.0461	1.1958e-05***	5	25699752	NCKAP1L
	ARS-BFGL-NGS-43909	−0.2359	0.0540	1.2275e-05***	5	30879776	
	ARS-BFGL-NGS-7725	−0.1716	0.0393	1.2868e-05***	5	27839649	KRT7
	ARS-USMARC-675	0.2939	0.0675	1.3141e-05***	5	31164990	ADCY6
	ARS-BFGL-NGS-8796	0.1810	0.0419	1.5218e-05***	5	29095603	
	ARS-BFGL-NGS-30168	0.1769	0.0423	2.8281e-05**	5	111313740	SYNGR1
	ARS-BFGL-NGS-717	0.1750	0.0419	3.0283e-05**	5	32861996	
	ARS-BFGL-NGS-98156	0.1705	0.0412	3.5276e-05**	5	27869236	
	Hapmap51043-BTA-73218	0.1580	0.0385	4.0667e-05**	5	29622395	LARP4
	ARS-USMARC-652	0.2198	0.0538	4.3781e-05**	5	45807839	
	Hapmap23022-BTA-161235	−0.1681	0.0417	5.4927e-05**	5	31613026	
	BTA-73209-no-rs	0.1581	0.0398	7.2288e-05*	5	29496625	DIP2B
	ARS-BFGL-NGS-100971	0.2301	0.0584	8.2505e-05*	5	28153566	
	ARS-BFGL-NGS-7567	−0.1945	0.0496	8.6988e-05*	5	28331294	SCN8A
	Hapmap56154-rs29018650	0.3068	0.0790	1.0251e-04*	5	35234997	
	Hapmap41950-BTA-72999	−0.1661	0.0438	1.4702e-04*	5	26082666	
	ARS-BFGL-NGS-68582	−0.1633	0.0437	1.8874e-04*	5	111380409	
	ARS-BFGL-NGS-11851	0.1600	0.0433	2.1791e-04*	5	32426908	
	ARS-BFGL-NGS-113311	−0.1585	0.0434	2.5842e-04*	5	27633265	KRT75
	BTA-73392-no-rs	0.1591	0.0437	2.7258e-04*	5	32104484	
	UA-IFASA-2781	0.1520	0.0419	2.8906e-04*	5	29989860	ASIC1
	ARS-BFGL-NGS-29931	−0.1441	0.0403	3.4662e-04*	5	36528436	TMEM117
	ARS-BFGL-NGS-10291	0.1635	0.0461	3.8575e-04*	5	24512405	TMCC3
	BTA-28787-no-rs	0.1471	0.0419	4.4336e-04*	5	37925443	
	BTB-01205531	−0.1641	0.0473	5.2633e-04*	5	33762720	
	ARS-BFGL-NGS-115973	−0.2631	0.0760	5.3487e-04*	5	28757093	
	ARS-BFGL-NGS-5790	−0.1463	0.0424	5.6436e-04*	5	26986116	
	ARS-BFGL-NGS-55084	−0.1462	0.0426	6.0193e-04*	5	60513092	
	Hapmap46916-BTA-105154	−0.1493	0.0439	6.7386e-04*	5	33246646	PCED1B
	ARS-BFGL-NGS-77906	−0.1452	0.0428	6.8482e-04*	5	24533402	TMCC3

Trait	SNP	effect	se	*p*-value	chr	bp	gene
	BTB-01495858	−0.1524	0.0451	7.3444e-04*	5	33981125	
	ARS-USMARC-Parent-DQ500958-no-rs	−0.1556	0.0461	7.4239e-04*	5	27825118	
	ARS-BFGL-NGS-33993	0.1379	0.0409	7.4239e-04*	5	107031270	TSPAN11
	ARS-BFGL-NGS-34352	0.1401	0.0417	7.7091e-04*	5	49842685	SRGAP1
	BTA-54940-no-rs	−0.1352	0.0404	8.1358e-04*	5	55263796	
	BTB-01205481	−0.1593	0.0479	8.7264e-04*	5	33808700	
	ARS-BFGL-NGS-34382	0.2680	0.0808	9.1605e-04*	5	26661043	ATF7
	ARS-BFGL-NGS-21191	−0.2432	0.0743	1.0598e-03*	5	26263552	
	ARS-BFGL-NGS-119788	−0.1809	0.0552	1.0656e-03*	5	30185840	
	Hapmap47087-BTA-73116	−0.2367	0.0726	1.1127e-03*	5	27898166	KRT80
	Hapmap39286-BTA-73191	−0.1373	0.0422	1.1248e-03*	5	30275164	
	ARS-BFGL-NGS-112163	−0.1323	0.0408	1.1809e-03*	5	31074760	
	ARS-BFGL-NGS-104808	−0.1517	0.0473	1.3231e-03*	5	111635921	
	ARS-BFGL-NGS-72504	−0.1684	0.0526	1.3595e-03*	5	24605672	
	Hapmap33270-BTA-75139	0.2224	0.0703	1.5654e-03*	5	112559317	
	ARS-BFGL-NGS-66566	−0.1534	0.0488	1.6800e-03*	5	36122701	
	ARS-BFGL-NGS-89539	0.1371	0.0436	1.6800e-03*	5	50175236	
	BTA-72912-no-rs	−0.1927	0.0614	1.6891e-03*	5	22020313	
	Hapmap41346-BTA-72920	0.1378	0.0441	1.7642e-03*	5	22196364	
	BTA-73296-no-rs	−0.1405	0.0450	1.7738e-03*	5	26526934	
	ARS-BFGL-NGS-93481	0.1565	0.0501	1.7738e-03*	5	115223183	
	Hapmap51042-BTA-73045	0.1642	0.0526	1.7835e-03*	5	24579644	
	ARS-BFGL-NGS-101539	0.1642	0.0526	1.7835e-03*	5	24628072	
	Hapmap24085-BTA-143102	−0.2800	0.0900	1.7835e-03*	5	46364432	
	ARS-BFGL-NGS-111020	−0.1231	0.0396	1.8730e-03*	5	106558376	
	ARS-BFGL-NGS-38038	−0.1840	0.0594	1.9671e-03*	5	27992179	NR4A1
	BTA-75143-no-rs	−0.1369	0.0444	2.0104e-03*	5	112647134	XPNPEP3
	ARS-BFGL-NGS-52457	−0.1403	0.0455	2.0660e-03*	5	104116518	PIANP
	BTB-02015191	0.1272	0.0413	2.0772e-03*	5	36553084	TMEM117
	BTA-73685-no-rs	0.1277	0.0415	2.0886e-03*	5	60929878	
	Hapmap41340-BTA-71012	−0.1602	0.0523	2.1937e-03*	5	115693793	
	ARS-BFGL-NGS-72724	−0.1150	0.0378	2.3167e-03*	5	116005043	

Trait	SNP	effect	se	*p*-value	chr	bp	gene
	ARS-BFGL-NGS-40375	−0.1277	0.0419	2.3294e-03*	5	36041533	NELL2
	ARS-BFGL-NGS-118817	−0.1889	0.0621	2.3421e-03*	5	31739928	
	BTB-00234784	−0.1331	0.0439	2.4334e-03*	5	88311948	ST8SIA1
	BTB-00225371	−0.1252	0.0414	2.5008e-03*	5	36578127	TMEM117
	Hapmap55179-rs29024483	0.1749	0.0428	4.3781e-05**	13	38830375	DZANK1
	Hapmap33865-BES2_Contig389_1251	−0.2103	0.0496	2.1860e-05**	29	13959142	

***5% genome-wide false discovery rate; **10% genome-wide false discovery rate; *5% chromosome-wide false discovery rate
